# Subinhibitory Concentrations of Bacteriostatic Antibiotics Induce *relA*-Dependent and *relA*-Independent Tolerance to β-Lactams

**DOI:** 10.1128/AAC.02173-16

**Published:** 2017-03-24

**Authors:** Pavel Kudrin, Vallo Varik, Sofia Raquel Alves Oliveira, Jelena Beljantseva, Teresa Del Peso Santos, Ievgen Dzhygyr, Dominik Rejman, Felipe Cava, Tanel Tenson, Vasili Hauryliuk

**Affiliations:** a University of Tartu, Institute of Technology, Tartu, Estonia; b Department of Molecular Biology, Umeå University, Umeå, Sweden; c Laboratory for Molecular Infection Medicine Sweden, Umeå University, Umeå, Sweden; d Institute of Organic Chemistry and Biochemistry, Czech Academy of Sciences, v.v.i., Prague, Czech Republic

**Keywords:** β-lactam, RelA, antibiotics, mupirocin, persistence, ppGpp, ribosomes, thiostrepton, tolerance, trimethoprim

## Abstract

The nucleotide (p)ppGpp is a key regulator of bacterial metabolism, growth, stress tolerance, and virulence. During amino acid starvation, the Escherichia coli (p)ppGpp synthetase RelA is activated by deacylated tRNA in the ribosomal A-site. An increase in (p)ppGpp is believed to drive the formation of antibiotic-tolerant persister cells, prompting the development of strategies to inhibit (p)ppGpp synthesis. We show that in a biochemical system from purified E. coli components, the antibiotic thiostrepton efficiently inhibits RelA activation by the A-site tRNA. In bacterial cultures, the ribosomal inhibitors thiostrepton, chloramphenicol, and tetracycline all efficiently abolish accumulation of (p)ppGpp induced by the Ile-tRNA synthetase inhibitor mupirocin. This abolishment, however, does not reduce the persister level. In contrast, the combination of dihydrofolate reductase inhibitor trimethoprim with mupirocin, tetracycline, or chloramphenicol leads to ampicillin tolerance. The effect is independent of RelA functionality, specific to β-lactams, and not observed with the fluoroquinolone norfloxacin. These results refine our understanding of (p)ppGpp's role in antibiotic tolerance and persistence and demonstrate unexpected drug interactions that lead to tolerance to bactericidal antibiotics.

## INTRODUCTION

Bacteria use an array of molecular systems to sense their environment and respond accordingly. The modulation of intracellular concentrations of alarmone nucleotides pppGpp and ppGpp, collectively referred to as (p)ppGpp, is one such system (1). An acute increase in (p)ppGpp levels upon stress—the so-called stringent response—drives the reallocation of available metabolic resources, gearing up bacterial physiology for stress resistance and survival. This regulatory system is of significant medicinal importance; (p)ppGpp plays a key role in the regulation of bacterial virulence (2) and contributes to bacterial survival during antibiotic treatment by both increasing the antibiotic tolerance of the bacterial population as a whole (3, 4) and driving the formation of a small subpopulation of highly tolerant cells, the so-called persister cells (5–7), in a generally sensitive culture. Therefore, (p)ppGpp signaling is a promising target for antibacterial drug development.

Cellular synthesis and degradation of (p)ppGpp is mediated by the members of the RelA/SpoT homolog (RSH) protein family, which are subdivided into two classes: “long” multidomain and “short” single-domain RSHs (8). In Escherichia coli two proteins, RelA and SpoT, represent the “long” RSHs. SpoT is a bifunctional protein, which can both synthesize and degrade (p)ppGpp and serves as a “hub” that integrates numerous stress signals and maintains the basal levels of the alarmone (9). RelA, also referred to as “the stringent factor,” has only one enzymatic activity, (p)ppGpp synthesis, and is specialized for the rapid response to a specific stress signal, amino acid starvation (10, 11). RelA is a ribosome-associated protein that works at the interface of active protein biosynthesis and ribosomal stalling in the presence of “hungry” codons, i.e., codons that are not efficiently decoded by cognate aminoacylated tRNAs due to amino acid shortage. It directly inspects the aminoacylation status of the incoming tRNA molecule in the ribosomal A-site (12–14) and, upon recognition of deacylated tRNA, i.e., lacking an amino acid attached to the 3′ CCA end, (p)ppGpp production by the enzyme is dramatically activated (10, 15). Conversely, active translation inhibits RelA via direct competition with translational factors, such as EF-G, and charged tRNA that does not activate RelA (10, 15, 16). The taxonomic distribution of RelA and SpoT is limited to Betaproteobacteria and Gammaproteobacteria, while the majority of bacterial species, including probably the second-best studied bacterial model organism, the firmicute Bacillus subtilis, possess a single “long” bifunctional RSH, Rel (8). Like RelA, it is a ribosome-associated factor, and Rel's synthetic activity is activated by deacylated tRNA (17). However, like SpoT (and unlike RelA), Rel is capable of hydrolyzing (p)ppGpp (17). The “short” RSH proteins are single-domain proteins that either synthesize (small alarmone synthetase [SAS]) or degrade (small alarmone hydrolase [SAH]) (p)ppGpp. E. coli lacks SAS, while in B. subtilis these proteins are represented by two enzymes, RelQ and RelP (18); both bacterial species lack SAHs (8). In response to stress conditions, the activity of SASs is regulated on the transcriptional level (10, 18), as well as via activation by (p)ppGpp (19, 20).

(p)ppGpp-mediated signaling is a promising target for the development of antibacterial agents since, first, this regulatory mechanism plays a central role in bacterial virulence and tolerance to antibiotics and, second, the (p)ppGpp-mediated cytoplasmic stringent response is absent in eukaryotes (21, 22). Several compounds targeting the stringent response have been developed in recent years. These molecules were suggested to act either via direct inhibition of RSHs, such as the (p)ppGpp analogue Relacin (21), or via catalytic hydrolysis of (p)ppGpp, such as antibiofilm peptide 1018 and its derivatives (22, 23). However, our follow-up studies have shown that neither Relacin nor peptide 1018 specifically inhibits the stringent response in live cells (24, 25).

An alternative strategy for inhibition of the stringent response is to take advantage of the intimate connection between the stringent response and ribosomal protein biosynthesis and to exploit existing antibiotics that target bacterial protein biosynthesis. The cyclic peptide thiostrepton is an efficient inhibitor of both translational GTPases, targeting initiation factor IF2 and elongation factors EF-Tu and EF-G on the ribosome (26–28), and E. coli RelA (at least in the test tube [29, 30]). This antibiotic intercalates between helices 43 and 44 of 23S rRNA and the ribosomal protein L11 (31). The latter is indispensable for the functionality of RelA (32), while the activity of EF-G is only moderately affected by the removal of L11 (33). The antibiotic tetracycline inhibits translation by precluding the accommodation of the A-site tRNA (34). Since binding of deacylated tRNA to the A-site is a prerequisite for the activation of RelA during amino acid starvation, it has been suggested that tetracycline can act as an indirect RelA inhibitor (30, 35). Moreover, all antibiotics targeting protein biosynthesis are expected to inhibit the RelA-mediated stringent response indirectly: inhibition of translation decreases the consumption of amino acids, which leads to an increase in the tRNA aminoacylation level. The prime example of this mechanism is seen with the antibiotic chloramphenicol, which is often used as a convenient tool for stringent response inhibition due to its fast uptake (36, 37).

In this report we reexamined the connections among antibiotic treatment, (p)ppGpp accumulation, antibiotic tolerance, and persistence. Using an E. coli *in vitro* translation and stringent response system assembled from individual purified components, we compared inhibition of the stringent factor RelA and translocation factor EF-G by the antibiotics thiostrepton, tetracycline, and chloramphenicol. We have contrasted our biochemical data with the effects of antibiotics in bacterial cultures by measuring bacterial growth and nucleotide pools using a high-pressure liquid chromatography (HPLC)-based approach. Finally, to put the results into an infection-relevant perspective, we tested the effects of the inhibition of the RelA-mediated stringent response by antibiotics on E. coli susceptibility to the β-lactam antibiotic ampicillin, which targets the cell wall, and to the fluoroquinolone antibiotic norfloxacin, which targets topoisomerase.

## RESULTS

### Thiostrepton specifically inhibits RelA activation by A-site deacylated tRNA while not affecting RelA activation by the ribosome itself.

Before proceeding with experiments, we had to address the problem of thiostrepton's tendency for precipitation. Using dynamic light scattering as a readout of precipitation, we tested the effects of addition of two commonly used organic solvents (dimethyl sulfoxide [DMSO] and 2,2,2-trifluoroethanol [TFE], both at 3% [wt/vol]) and a hydrophilic nonionic surfactant, Poloxamer 407 (trademarked by BASF as Pluronic F-127) at 0.1% (wt/vol). While the addition of DMSO and TFE has only a modest effect on thiostrepton's solubility, Pluronic F-127 keeps the antibiotic in solution at concentrations up to 15 μM (see Fig. S3A in the supplemental material). We have validated that the addition of the surfactant did not interfere with the *in vitro* system by monitoring the 70S-dependent activity of translational GTPase EF-G (see Fig. S3B in the supplemental material) and the stringent response factor RelA (see Fig. S3C in the supplemental material).

**FIG 1:**
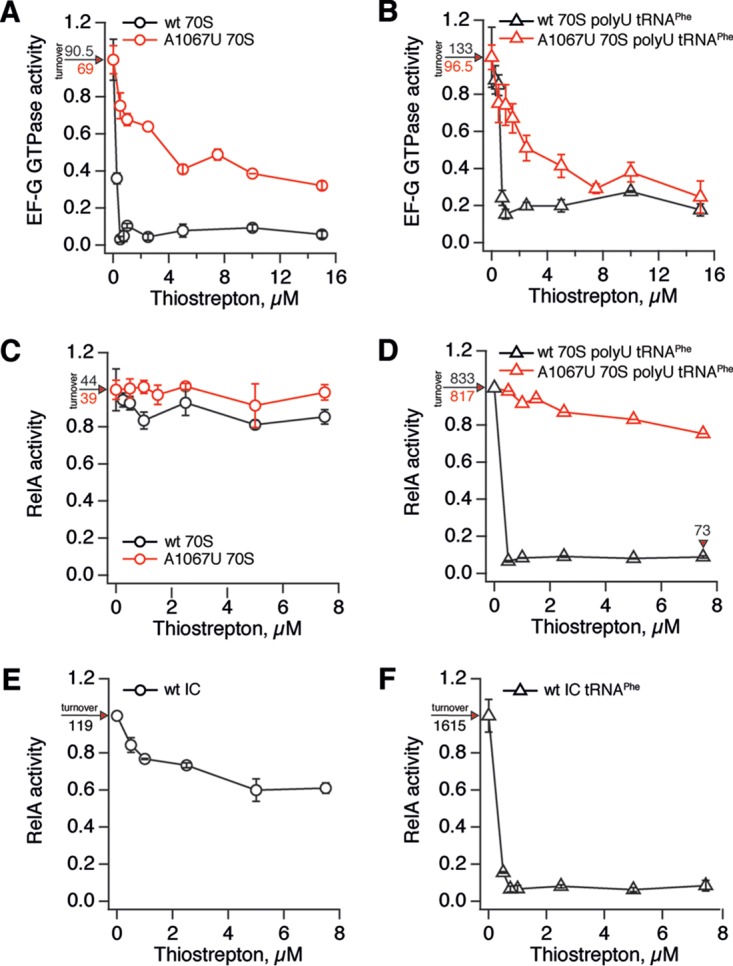
Thiostrepton specifically inhibits RelA activation by deacylated A-site tRNA. EF-G GTPase is efficiently inhibited by thiostrepton in the presence of vacant wild-type 70S ribosomes (A) or starved ribosomal complexes (B). A1067U rRNA mutation protected EF-G from thiostrepton in both cases (A and B). RelA activated by either vacant wild-type or A1067U 70S ribosomes (C) or initiation complexes (E) is insensitive to thiostrepton. RelA activated by 70S ribosomes programmed with 2 μM poly(U) mRNA and 2 μM deacylated tRNA^Phe^ is efficiently inhibited by thiostrepton, and rRNA mutation A1067U protects from antibiotic (D). Similarly, efficient inhibition is observed when RelA is activated by ribosomal initiation complexes in the presence of 2 μM deacylated A-site tRNA^Phe^ (F). The enzymatic activities of 100 nM elongation factor EF-G (GTP hydrolysis) and 100 nM stringent factor RelA were assayed in the presence of 0.5 μM 70S or wild-type initiation complexes (black traces) or thiostrepton-resistant A1067U rRNA mutant ribosomes (red traces). Enzymatic activities (turnovers, GTP per EF-G per minute and ppGpp per RelA per minute) were normalized to that of the corresponding system in the absence of thiostrepton, and uninhibited turnover values corresponding to 1.0 activity are provided on individual panels. Error bars represent the standard deviations of the turnover estimates as determined by linear regression. Each experiment was performed at least three times.

After establishing the conditions under which thiostrepton is soluble in a wide range of concentrations, we characterized RelA inhibition by the antibiotic using an *in vitro* stringent response system (38). We used two specificity controls. First, we took advantage of thiostrepton-resistant ribosomes carrying an A1067U mutation in the 23S rRNA (39). Second, we monitored the inhibition of the GTPase activity of translocase EF-G, a well-studied molecular target of thiostrepton. GTP hydrolysis by EF-G was efficiently inhibited by thiostrepton both when the experiment was performed in the presence of vacant 70S ribosomes (Fig. 1A) and when 70S ribosomes were programmed with poly(U) model mRNA and deacylated tRNA^Phe^ (Fig. 1B). At the same time in both experimental systems, EF-G activation by A1067U ribosomes is far less sensitive to thiostrepton, an observation in good agreement with earlier reports (39). Unlike EF-G, weak induction of RelA's enzymatic activity by vacant ribosomes is virtually insensitive to thiostrepton (Fig. 1C). However, the situation changes dramatically when RelA is activated by ribosomes programmed poly(U) mRNA and deacylated tRNA^Phe^ (Fig. 1D). In this system RelA is almost 30 times more active than in the presence of empty 70S ribosomes, which is consistent with a role of deacylated tRNA in the activation of RelA (10). Thiostrepton efficiently inhibits RelA, although the inhibition is incomplete. The turnover rate of the remaining thiostrepton-insensitive ppGpp synthesis is similar to that observed in the absence of deacylated tRNA^Phe^ (73 versus 44 ppGpp per RelA per min). The A1067U rRNA mutation results in near-complete immunity to thiostrepton, a stronger effect than that observed in the case of EF-G (compare Fig. 1B and D). We confirmed the strict tRNA dependence of RelA inhibition by thiostrepton observed in the poly(U)-driven system using a more physiologically relevant model system, i.e., RelA activated by ribosomal initiation complexes programmed with model mRNA with an open reading frame coding for the MF dipeptide (Fig. 1E and F).

Next, we tested whether the dependence of RelA inhibition by thiostrepton on the presence of A-site deacylated tRNA, the ultimate inducer of the enzyme's enzymatic activity (10, 35) and a key factor in promoting RelA binding to the ribosome (40), is specific to this antibiotic. We have tested antibiotics tetracycline and chloramphenicol that inhibit translation and have been reported to abrogate RelA-mediated ppGpp accumulation in live bacteria (30, 35–37). Inhibition by tetracycline is rather inefficient and, somewhat surprisingly, only mildly more pronounced in the presence of deacylated A-site tRNA (see Fig. S4A in the supplemental material). Chloramphenicol, while being a potent inhibitor of translation, has no effect on RelA (see Fig. S4B in the supplemental material).

### Inhibition of translation by antibiotics blocks the RelA-mediated stringent response in live cells.

**FIG 2:**
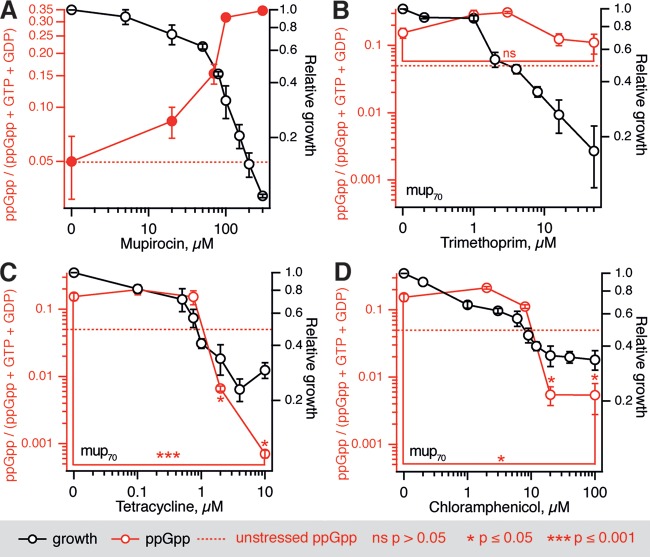
Concurrent inhibition of E. coli growth and ppGpp production by antibiotics targeting translation. (A) A stringent response was induced by the addition of increasing concentrations of mupirocin, followed by 30 min of incubation and HPLC analysis. (B to D) Cell cultures were treated for 30 min with increasing concentrations of trimethoprim (B), tetracycline (C), or chloramphenicol (D) combined with 70 μM mupirocin (mup_70_), samples were collected, and nucleotide levels were determined by HPLC. Experiments were performed with BW25113 E. coli wild-type strain grown at 37°C in MOPS medium supplemented with 0.4% glucose and a full set of 20 amino acids at 25 μg/ml. Growth inhibition was calculated as an increase in the OD_600_ after 1 h of antibiotic treatment compared to the untreated control. The ppGpp levels are calculated as a ppGpp fraction of a combined GTP, GTP, and ppGpp nucleotide pool; the dashed red trace indicates the level in unstressed cells. Error bars indicate the standard errors of the mean (three to five biological replicates). The *P* values were calculated using a two-tailed Welch's *t* test either relative to the unstressed ppGpp levels or, where indicated by brackets, within the titration series.

To put our biochemical results in the context of bacterial physiology, we characterized the effects of translational inhibitors on the intracellular levels of nucleotides ppGpp, GTP, GDP, and ATP in E. coli and B. subtilis using an HPLC-based approach. These two common model organisms represent the two archetypical regulatory architectures of the stringent response system; E. coli relies on direct regulation of RNAP by ppGpp (41), while in B. subtilis ppGpp synthesis effectuates changes in the transcriptional program indirectly via consumption of GTP (42), affecting the ratio of GTP and ATP levels which, in turn, is sensed by RNAP (43). We contrasted the effects of three antibiotics specifically targeting protein biosynthesis—thiostrepton, chloramphenicol, and tetracycline—with that of trimethoprim, an antimetabolite antibiotic that blocks the production of tetrahydrofolate by dihydrofolate reductase, resulting in the inhibition of glycine, methionine, dTTP, and purine biosynthesis (44). To induce (p)ppGpp accumulation, we pretreated exponentially growing bacterial cultures with a competitive inhibitor of the isoleucyl-tRNA synthetase antibiotic mupirocin (pseudomonic acid). This treatment dramatically increases the amount of deacylated tRNA in the cell, leading to activation of RelA/Rel and triggering an acute stringent response (45) (Fig. 2A).

**FIG 3:**
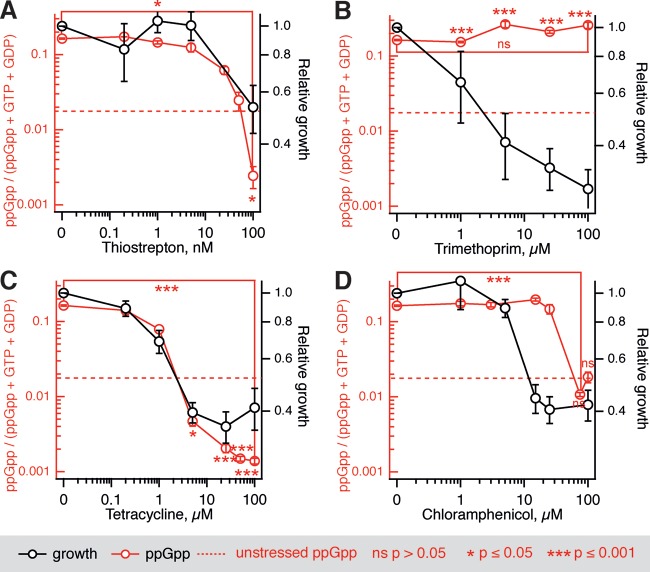
Inhibition of growth of B. subtilis artificially starved for isoleucine by antibiotics targeting translation is mirrored by a drop in ppGpp levels. Artificial starvation for isoleucine induced by addition of 70 nM mupirocin (mup_70_) was countered by increasing concentrations of thiostrepton (A), trimethoprim (B), tetracycline (C), or chloramphenicol (D). At 30 min after the addition of antibiotics, samples were collected, and the nucleotide levels were determined by HPLC. Experiments were performed with BSB1 B. subtilis wild-type strain grown at 37°C in MOPS medium supplemented with 0.4% glucose and a full set of 20 amino acids at 25 μg/ml. The ppGpp levels were calculated as a ppGpp fraction of a combined GTP, GTP, and ppGpp nucleotide pool; the dashed red trace indicates the level in unstressed cells. Error bars indicate the standard errors of the mean (three to five biological replicates). The *P* values were calculated using a two-tailed Welch's *t* test either relative to the unstressed ppGpp levels or, where indicated by brackets, within the titration series.

All of the antibiotics targeting translation tested in the biochemical assays also inhibit mupirocin-induced ppGpp accumulation both in E. coli (BW25113 wild-type strain) (Fig. 2) and B. subtilis (BSB1 wild-type strain) (Fig. 3). Since E. coli is insensitive to thiostrepton *in vivo* due to a lack of cellular uptake (46), experiments with this antibiotic could be performed only with B. subtilis. With the exception of B. subtilis treated with chloramphenicol, complete growth inhibition by three translation-targeting antibiotics causes a statistically significant drop in the levels of ppGpp below the level in unstressed cells. Importantly, inhibition of growth by trimethoprim does not lead to a decrease in the ppGpp levels in either E. coli or B. subtilis (Fig. 2B and 3B).

The ratio between the levels of GTP and ATP, rather than the (p)ppGpp levels, is the key effector of the stringent response in B. subtilis (43). The concentrations of the two triphosphate species change reciprocally during the stringent response (47), regulating the transcriptional program via initiator NTP concentrations (43). In the absence of additional antibiotics, pretreatment of the wild-type B. subtilis BSB1 strain with 70 nM mupirocin in order to induce the stringent response leads to an almost 3-fold change in the GTP/ATP ratio (Table 1; see also Fig. S5 in the supplemental material). The ratio of GTP and ATP concentrations in B. subtilis pretreated with mupirocin readily increases with the addition of increasing concentrations of translational antibiotics tetracycline and chloramphenicol, surpassing the unstressed levels at the concentrations causing complete inhibition of growth. Conversely, the GTP/ATP ratio is nearly insensitive to the addition of trimethoprim and does not change even upon the complete inhibition of growth by a 100 μM concentration of antibiotic. The GTP levels are dramatically elevated in a ppGpp^0^ B. subtilis strain, i.e., a strain lacking functional RSH enzymes (48). Our data show that treatment with antibiotics inhibiting protein synthesis leads to a similar effect.

**TABLE 1:** GTP/ATP ratios in B. subtilis upon antibiotic treatment^*a*^

Condition	Mean GTP/ATP ratio	SD	SEM
No treatment	0.54	0.24	0.03
Mup_70 nM_	0.18	0.04	0.05
Mup_70 nM_ Thio_100 nM_	1.12	0.72	0.24
Mup_70 nM_ Tet_100 μM_	1.51	0.66	0.13
Mup_70 nM_ Cam_100 μM_	0.55	0.35	0.12
Mup_70 nM_ Trim_100 μM_	0.21	0.06	0.01

a Artificial starvation for isoleucine induced by addition of 70 nM mupirocin was countered by the secondary antibiotic challenge. At 30 min after the addition of antibiotics, samples were collected, and the nucleotide levels were determined by HPLC. Experiments were performed with BSB1 B. subtilis wild-type strain grown at 37°C in MOPS medium supplemented with 0.4% glucose and a full set of 20 amino acids at 25 μg/ml. Mup, mupirocin; Thio, thiostrepton; Tet, tetracycline; Cam, chloramphenicol; Trim, trimethoprim.

### Antibiotic pretreatment induces *relA*-dependent and -independent tolerance to the β-lactam ampicillin but not to the fluoroquinolone norfloxacin.

Upon establishing that antibiotics targeting protein biosynthesis efficiently inhibit the stringent response, we proceeded to scrutinize the effects of inhibition of translation, and by proxy ppGpp accumulation, on tolerance to bactericidal antibiotics and antibiotic persistence. We use the terms tolerance and persistence according to the definitions of Brauner et al. (49): “tolerance” is the slower killing of bacterial population as a whole upon exposure to bactericidal antibiotic, whereas “persistence” is mediated by a small, slowly killed subpopulation in a generally rapidly killed culture and is manifested in biphasic killing kinetics (see Fig. S1 in the supplemental material).

We have used three E. coli K-12 strains: (i) a wild-type strain harboring native functional *relA* and *spoT* genes (BW25113), (ii) an isogenic “relaxed” strain with the *relA* gene deleted by the homologous recombination method of Datsenko and Wanner (50), and (iii) an isogenic strain lacking not only *relA* but also *spoT*, a less enzymatically efficient (p)ppGpp synthetase regulated by many inputs (9). The latter strain is completely devoid of ppGpp, the so-called ppGpp^0^ phenotype, and it was this type of strain that was originally used to propose the connection between elevated (p)ppGpp levels and persistence (6, 51, 52).

**FIG 4:**
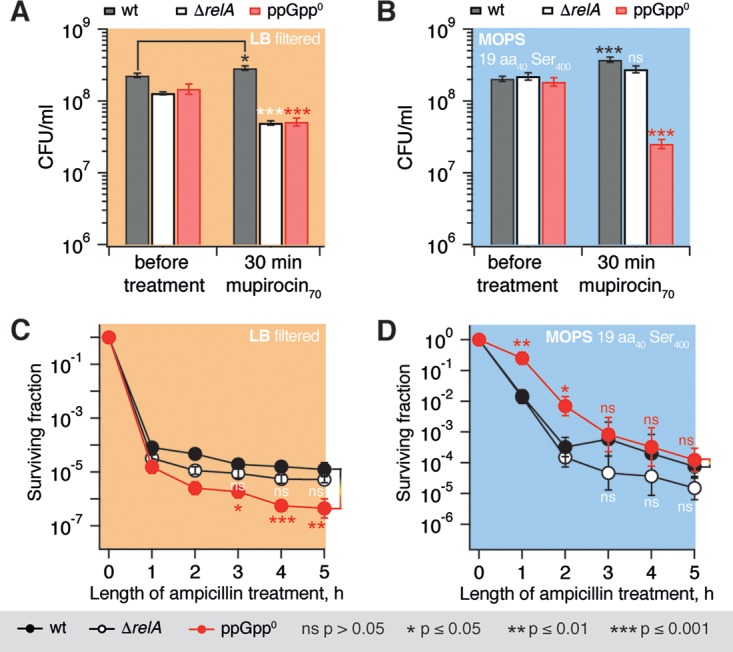
Medium composition modulates the effects of mupirocin and ampicillin treatment on wild-type, relaxed, and ppGpp^0^ BW25113 E. coli. Experiments were performed using either filtered LB (A and C; beige shading) or MOPS medium supplemented with 0.4% glucose and a full set amino acids at 400 μg/ml (for serine) or 25 μg/ml (for the remaining 19 amino acids) (B and D; blue shading). The colony count (i.e., the CFU) was determined either as a function of a 30-min treatment with either 70 μM mupirocin compared to mock treatment (A and B) or as a function of time after the addition of ampicillin to a final concentration of 200 μg/ml (C and D). Error bars indicate the standard errors of the mean (three to five biological replicates). Where indicated by brackets, the *P* values were calculated using a two-tailed Welch's *t* test either between wild-type and mutant strains (A and B) or relative to the killing time course of an untreated culture (C and D).

Our previous investigations of antibiotic tolerance of relaxed E. coli BW25113 have used the synthetic defined medium M9 supplemented with 0.4% glucose (53). However, the original report connecting (p)ppGpp and persistence in E. coli mostly used lysogeny broth (LB) medium (51). LB is a complex medium in which bacteria successfully adjust their physiology to several shifts of limiting nutrients along the growth curve (54, 55). This can be problematic for investigations of bacterial physiology (56), especially given that persistence can be highly sensitive to media composition (57). Therefore, we compared the growth and ampicillin killing kinetics of wild-type, relaxed, and ppGpp^0^ strains in LB and morpholinepropanesulfonic acid (MOPS) media. In LB the growth curves of both wild-type and ppGpp^0^ strains are nearly indistinguishable (see Fig. S6A in the supplemental material). However, in MOPS medium supplemented with 0.4% glucose and a full set of 20 amino acids at 25 μg/ml (the very conditions we used for nucleotide measurements), the growth of the ppGpp^0^ strain halts already at an optical density at 600 nm (OD_600_) of 0.01, whereas the relaxed strain shows no pronounced growth defect (see Fig. S6B in the supplemental material). In accordance with a study by Potrykus et al. (58), we increased the serine concentration to 400 μg/ml in our MOPS-based medium, which nearly eliminated the relative growth defect of the ppGpp^0^ strain: the growth rate during the exponential stage matches that of the wild type, but the ppGpp^0^ strain still enters the stationary phase at a somewhat lower OD_600_ (see Fig. S6C in the supplemental material). Next, we tested whether the tool used for the induction of the stringent response, i.e., the Ile-RS inhibitor mupirocin, by itself has an effect on bacterial viability. In good agreement with earlier reports stating that mupirocin is a bacteriostatic antibiotic (59), there is no CFU loss in wild type after 30 min of treatment with 70 μM mupirocin in either of the two media (Fig. 4A and B). The antibiotic concentration was chosen so that it decreases the growth rate approximately twice and induces a half-maximal increase in ppGpp (Fig. 2A). However, in the case of the relaxed strain, the colony count drops around 1 order of magnitude upon mupirocin treatment in LB, but not in MOPS supplemented with serine; the ppGpp^0^ strain loses around 1 log of CFU under both conditions. Next, we monitored the ampicillin killing kinetics of the three strains in LB (Fig. 4C) and serine-supplemented MOPS (Fig. 4D). The loss of *relA* does not have a statistically significant effect on persister count (i.e., CFU count at the 5-h time point) in either of the two media. The simultaneous loss of *spoT* and *relA* results in a moderate, yet statistically significant 10-fold decrease in persister count in LB; in MOPS medium the persister count is not affected. In MOPS, however, the ppGpp^0^ strain has moderately increased ampicillin tolerance (i.e., slower ampicillin killing of the bulk of bacterial population). The likely explanation for this is a moderate growth defect of the mutant since the rate of ampicillin killing is proportional to the growth rate (60).

We decided to use only relaxed and wild-type strains in the following experiments and to omit the ppGpp^0^ strain since (i) we could not optimize the defined medium so that there would not be any interference from growth rate and mupirocin effects present in the system already before we performed antibiotic treatments and (ii) since inhibition of *de novo* protein synthesis in a Δ*relA* background would either way render the cell essentially ppGpp^0^ since E. coli SpoT's synthetic activity was rapidly lost upon the inhibition of protein synthesis (61). We decided to adhere to the original version of MOPS, i.e., supplemented with 20 amino acids at 25 μg/ml without the additional serine, since, first, under these conditions wild-type and Δ*relA* strains grow similarly and, second, an overabundance of serine might cause perturbations in the metabolism of the relaxed strain (62).

We tested whether pretreatment with translational inhibitors chloramphenicol and tetracycline would affect E. coli tolerance to the β-lactam antibiotic ampicillin. To deconvolute specific antibiotic effects from growth rate effects, we used concentrations of antibiotics that reduce the growth rate by half (the experimental setup is outlined on Fig. S1 in the supplemental material; growth measurements are presented in Fig. S7 in the supplemental material). In the case of the wild-type BW25113 E. coli strain, the killing kinetics of cultures pretreated with chloramphenicol and tetracycline show a slight increase in both ampicillin tolerance and persistence compared to the untreated culture and to culture treated with trimethoprim. Increasing concentrations of translational inhibitors chloramphenicol and tetracycline roughly three times (to 20 and 2 μM, respectively) does not alter the killing kinetics (see Fig. S8 in the supplemental material), suggesting that the observed differences in killing kinetics can be attributed to the nature of the antibiotic challenge rather than to the extent of growth inhibition. Our results are in good agreement with previous observations that the arrest of protein biosynthesis leads to increased persister formation (63). Pretreatment with mupirocin, however, results in a greatly enhanced ampicillin tolerance of the wild-type strain. This effect was strictly dependent on the functionality of the *relA* gene, supporting the key role of the stringent response in bacterial persistence (5, 6, 51) and ampicillin tolerance (3, 64–67).

One could attribute the inability of chloramphenicol and tetracycline pretreatment to sensitize bacteria to ampicillin via inhibition of ppGpp production to already low intracellular ppGpp levels in rapidly growing cells prior to the ampicillin challenge: since low initial ppGpp levels could not protect the cells from ampicillin in the first place, lowering ppGpp further had no effect. In all our nucleotide measurements, an acute stringent response was initially induced by mupirocin, and then the ppGpp levels were affected by subsequent antibiotic treatments (Fig. 2 and 3).

**FIG 5:**
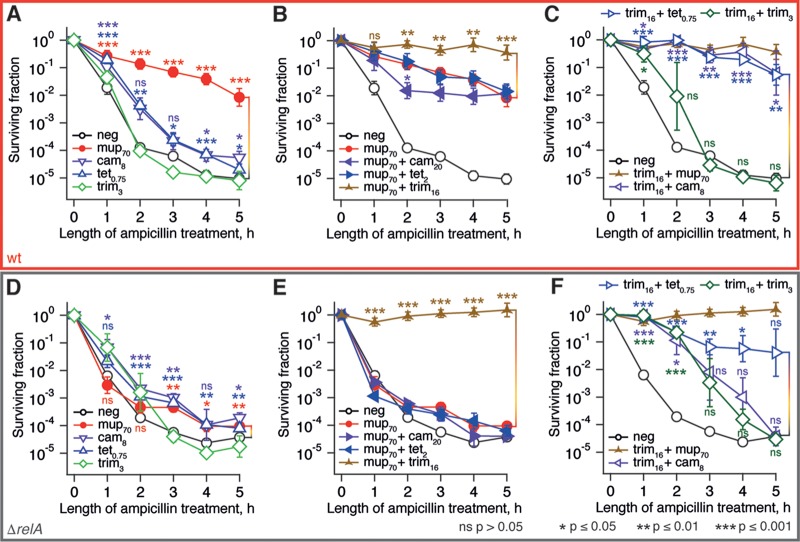
Antibiotic pretreatment induces both *relA*-dependent and *relA*-independent ampicillin tolerance of BW25113 E. coli. The antibiotic pretreatment was performed for 30 min at 37°C in MOPS medium supplemented with 0.4% glucose and amino acids at 25 μg/ml using BW25113 E. coli wild-type strain (A to C) and an isogenic *relA* knockout (Δ*relA*; D to F), followed by the addition of ampicillin to a final concentration of 200 μg/ml. The surviving fraction was determined by LB plating and colony counting. The bacteriostatic antibiotics were used at concentrations reducing the growth rate by half, and concentrations are indicated in μM on the figures, e.g., mup_70_ indicates pretreatment with 70 μM mupirocin. Error bars indicate the standard errors of the mean (three to five biological replicates). Where indicated by brackets, the *P* values were calculated by using a two-tailed Welch's *t* test relative to the killing time course of an untreated culture.

Therefore, we repeated the time-kill assays with bacterial cultures pretreated with mupirocin in a similar fashion (Fig. 5B and E). We used translational inhibitors at concentrations that repress ppGpp below the unstressed level and result in growth inhibition by ca. 80%; in the case of trimethoprim, we used a concentration (16 μM) that inhibits the growth rate to the same extent. When added at these concentrations (in the absence of mupirocin), neither of the antibiotics protects cells from ampicillin killing (see Fig. S8 in the supplemental material). Surprisingly, neither chloramphenicol nor tetracycline can efficiently abrogate mupirocin-induced RelA-dependent ampicillin tolerance. Moreover, treatment with a combination of mupirocin and trimethoprim results in complete tolerance to the ampicillin challenge, and this effect was equally pronounced in both stringent wild-type and relaxed E. coli strains (compare the traces labeled as brown, three-pointed stars in Fig. 5B and E). We observed no increase in ampicillin persistence (i.e., at 4- and 5-h time points) or statistically significant increases in tolerance (i.e., at the 1-h time point) upon pretreatment with trimethoprim alone in both the wild type and the relaxed strain. This suggests that *relA*-independent ampicillin tolerance is a result of the interaction between the effects of trimethoprim and mupirocin rather than an effect of either of the antibiotics. Since mupirocin is, in essence, a translational inhibitor, we tested whether the combination of trimethoprim with other translational inhibitors, i.e., chloramphenicol and tetracycline, results in the similar protective effect (Fig. 5C and F). In the wild-type strain, these combinations do, indeed, confer high levels of ampicillin tolerance throughout the whole 5-h killing experiment. In the relaxed strain the combination of trimethoprim and tetracycline has a strong protective effect, increasing both tolerance and persistence. Pretreatment with trimethoprim combined with chloramphenicol behaves similarly to the specificity control, i.e., addition of extra trimethoprim at 16 μM; while the pretreatment does decrease the initial killing rate (i.e., induces tolerance), it does not affect the plateau (i.e., does not increase persistence).

The observed combinatorial ampicillin tolerance could be explained by growth inhibition (60). However, growth inhibition does not correlate with tolerance (see Fig. S7 in the supplemental material). Alternatively, tolerance could be caused by ampicillin-tolerant Ldt enzymes overproducing DAP-DAP cross-links in the cell wall (68). However, although the mupirocin/trimethoprim combination does result in DAP-DAP accumulation, this is not essential for tolerance (see Fig. S9C in the supplemental material).

**FIG 6:**
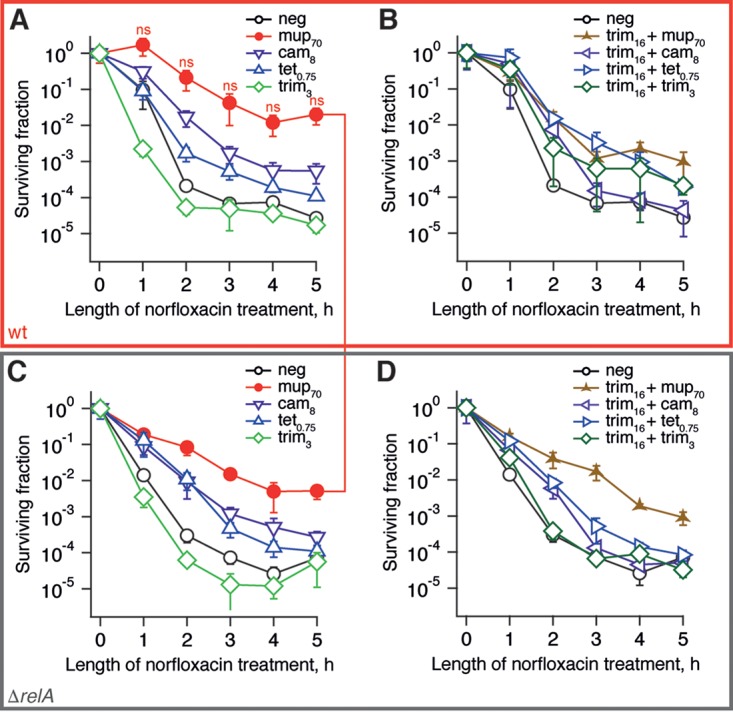
RelA functionality does not determine the norfloxacin tolerance of BW25113 E. coli induced by antibiotic pretreatment. The antibiotic pretreatment was performed for 30 min at 37°C in MOPS media supplemented with 0.4% glucose and amino acids at 25 μg/ml using BW25113 E. coli wild-type strain (A and B) and an isogenic *relA* knockout (Δ*relA*; C and D), followed by the addition of norfloxacin to a final concentration of 5 μg/ml, and the surviving fraction was determined by LB plating and colony counting. Bacteriostatic antibiotics were used at concentrations reducing the growth rate by half, and concentrations are indicated in μM on the figures, e.g., mup_70_ indicates pretreatment with 70 μM mupirocin. Error bars indicate the standard errors of the mean (three to five biological replicates). *P* values were calculated using a two-tailed Welch's *t* test between wild-type and relaxed strains.

Finally, in order to discriminate between general multidrug tolerance and specific tolerance to ampicillin, we performed the same set of time-kill experiments, substituting ampicillin for the fluoroquinolone norfloxacin (Fig. 6). The two sets of killing curves are profoundly different. First, in the norfloxacin set we see a very modest effect of *relA* disruption, suggesting that the functionality of the RelA-mediated stringent response is not crucial to antibiotic-induced tolerance to norfloxacin under the experimental conditions used. Second, the dramatic protective effect of the trimethoprim and mupirocin combination is absent in the case of the norfloxacin data set.

## DISCUSSION

In this report we characterized the effects of antibiotics targeting translation on (i) RelA-mediated (p)ppGpp synthesis and (ii) antibiotic persistence. Since (p)ppGpp is believed to be the primary driver behind the formation of persisters (5–7), one could expect that the inhibition of (p)ppGpp accumulation by antibiotics would decrease the persistence levels. We have been sequentially moving from a reductionist biochemical system to more biologically relevant measurements via the following steps: (i) biochemical studies of antibiotic effects on ppGpp production by E. coli stringent response factor RelA in the test tube; (ii) microbiological measurements of antibiotic effects on ppGpp production in E. coli and B. subtilis bacterial cultures; and, finally, (iii) microbiological characterization of the downstream effects of (p)ppGpp depletion on antibiotic tolerance of E. coli. As discussed below, in the course of this investigation we have made observations important for fields of antibiotic development, structure-functional investigations of the ribosome, antibiotic tolerance, and persistence.

### Efficient solubilization of thiostrepton by Pluronic F-127.

Thiostrepton is virtually insoluble in water and can only be dissolved in organic solvents, such as DMSO (46). Therefore, considerable effort has gone into the development of more soluble derivatives using chemical synthesis (69) or bioengineering approaches (70). Biochemical experiments with unmodified thiostrepton performed in aqueous buffers in the absence of solubilizing agents are at risk of potential artifacts (26). The addition of nonionic surfactant Pluronic F-127 to the reaction mixture dramatically increases thiostrepton's solubility and renders the antibiotic amenable to biochemical studies (see Fig. S3 in the supplemental material). Importantly, since micellation of F-127 is abrogated at low temperatures (71) experiments with F-127-solubulized thiostrepton should be performed at 30 to 37°C. F-127 is successfully used for the controlled delivery of several drug classes (72), and our results suggest its usefulness for topical formulations of thiostrepton.

### A-site tRNA-dependent inhibition of RelA by thiostrepton.

The peptide antibiotic thiostrepton has a subnanomolar affinity to its molecular target, the 50S ribosomal subunit (73). The drug intercalates between ribosomal protein L11 and helices H43 and H44 of the 23S rRNA, affecting the structure and conformational dynamics of the so-called GTPase-associated center, GAC: the ribosomal region responsible for binding and stimulation of the GTPase activity of translational factors (31). The ribosomal protein L11 contributes to the regulation of translational GTPases, such as EF-G (31, 74), and is essential for RelA activation on the ribosome (32). The A1067U mutation in H43 renders ribosomes highly resistant to thiostrepton (39), decreasing the antibiotic affinity by 1,000-fold (73).

We have shown that thiostrepton specifically inhibits RelA activation by the A-site tRNA and has no effect on RelA activated by 70S alone, whereas EF-G is inhibited equally efficiently regardless of the presence or absence of A-site tRNA (Fig. 1). The recent cryo-electron microscopy reconstructions of E. coli RelA complexed with a “starved” ribosomal complex provide a structural explanation (12–14). On the ribosome, RelA wraps around the A-site tRNA, distorting it and driving into a conformation similar to that of preaccommodated tRNA in complex with EF-Tu, the A/T state (75). In this conformation, nucleotide C56 of the A-site tRNA elbow forms a stacking interaction with rRNA residue A1067, the very residue crucial for thiostrepton binding. Interestingly, the A1067U thiostrepton-resistant mutation used in the present study does not affect RelA activity (Fig. 1C and D). In the absence of the A-site tRNA, the N-terminal catalytic domains of 70S-bound RelA are disordered and do not form stable contacts with the ribosome (14), providing a structural explanation for insensitivity to thiostrepton.

### Inhibition of protein synthesis efficiently represses (p)ppGpp accumulation.

Specific inhibition of RelA is not, however, necessary for efficient inhibition of the stringent response: all of the tested protein synthesis inhibitors indirectly inhibit an acute stringent response in both E. coli and B. subtilis (Fig. 2 and 3; see also Fig. S5 in the supplemental material). Although ribosomal antibiotics directly or indirectly target ribosome-dependent RSHs RelA (E. coli) or Rel (B. subtilis) that are responsible for the acute accumulation of (p)ppGpp under amino acid starvation, both of these organisms possess other sources of (p)ppGpp, namely, SpoT in E. coli (76) and two small alarmone synthetases (SASs), YjbM and YwaC in B. subtilis (18). However, since E. coli SpoT's synthetic activity is exceedingly unstable, i.e., it is lost within a minute upon inhibition of protein synthesis (61), inhibition of RelA in combination with that of protein synthesis is likely to render E. coli effectively unable to produce (p)ppGpp, i.e., ppGpp^0^.

### β-Lactam tolerance is induced by antibiotic pretreatment in the absence or presence of the RelA-mediated stringent response.

Efficient depletion of (p)ppGpp by antibiotics targeting translation has nontrivial effects on antibiotic tolerance and persistence. The dramatic difference in ampicillin and norfloxacin killing results (compare Fig. 5 and 6) supports the assertion that elevated levels of (p)ppGpp induce antibiotic tolerance via antibiotic-specific pathways rather than protection from antibiotics in general (57, 77–80). Although the role of (p)ppGpp in ampicillin tolerance is well established, (p)ppGpp's role in protection from gyrase inhibitors such as norfloxacin is controversial: although some studies, similar to our own results, report an absence of specific protection (81), other studies observed prominent effects (4, 82). The difference is likely to be attributable to differences in experimental conditions, such as medium composition; differences in mupirocin killing of the ppGpp^0^ E. coli strain in LB and MOPS media (Fig. 4) provide yet another example of a medium-specific effect.

Preexposure to trimethoprim combined with mupirocin induces a near-complete *relA*-independent tolerance to β-lactams ampicillin and imipenem (Fig. 5B and E; see also Fig. S9D and E in the supplemental material); substitution of mupirocin for other translational inhibitors, such as chloramphenicol or tetracycline, results in a weaker effect (Fig. 5C and F). Our results reinforce the already well-established connection between antibiotic pretreatment and induction of tolerance (83), specifically for bacteriostatic antibiotics (such as inhibitors of protein synthesis) that protect bacteria from bactericidal drugs (such as β-lactams) (84). Since in our experimental system the protection was specific for ampicillin, as opposed to norfloxacin, we hypothesize that the effect is connected to ampicillin's mode of action. However, at present we have been unable to determine its exact molecular mechanism, though it does not appear to be the result of accumulation of DAP-DAP cross-links (see Fig. S9 in the supplemental material).

### Inhibition of the ppGpp-mediated signaling: a promising drug target but not a universal solution to antibiotic persistence.

Several recent reports have documented the existence of antibiotic persistence in bacterial strains lacking (p)ppGpp-mediated signaling (85, 86). Rather than acting via (p)ppGpp signaling, in Staphylococcus aureus persistence is associated with a stationary-phase-like physiological state, characterized by low ATP levels and the expression of characteristic stationary-phase markers (86). Similarly, E. coli persister populations are enriched in bacteria with low metabolic activity (87). Our double-pronged challenge targeting both nucleotide synthesis and translation could be causing β-lactam tolerance in a similar fashion, despite the efficient inhibition of (p)ppGpp accumulation. Finally, the abrogation of the ppGpp-mediated signaling in E. coli via genetic disruption of *relA* and *spoT* results in medium-specific effects on tolerance and persistence (Fig. 4), again indicating that (p)ppGpp is not the one and only driver of persistence.

Therefore, we advocate a model with numerous parallel routes leading to persistence (88). (p)ppGpp, despite being important, is not the sole driver of the phenomenon. Specific inhibitors of the stringent response are unlikely to completely eradicate persistence. However, disrupting (p)ppGpp signaling will compromise virulence (89) and have profound effects on crucial aspects of amino acid (90) and nucleotide metabolism (42). Therefore, RSH enzymes are still a very promising drug target. Given recent progress in organic synthesis and biological engineering of thiopeptides (91), the thiostrepton molecular scaffold could lead to the development of specific and potent inhibitors of ribosome-associated RHS enzymes Rel and RelA.

## MATERIALS AND METHODS

### Dynamic light scattering analysis of thiostrepton solubility.

Serial dilutions of thiostrepton (Tocris; 1, 2, and 5 μM) were prepared in HEPES-Polymix buffer (25 mM HEPES [pH 7.5], 1 mM dithiothreitol, 15 mM Mg^2+^) (92, 93) supplemented with 3% TFE, 3% DMSO, or 0.1% (wt/vol) Pluronic F-127 (Sigma). Next, 50 μl aliquots were analyzed on Zetasizer Nano S90 (Malvern) in microcuvettes (Malvern).

### TLC analysis of nucleotide mixtures.

Both EF-G GTPase reaction and ppGpp synthesis by RelA were monitored by thin-layer chromatography (TLC) analysis of ^3^H-labeled nucleotides, followed by scintillation counting according to the method of Mechold et al. (94), with modifications. Time points from the reaction mixtures (5 μl; see below for details) were quenched by the addition 4 μl of 70% formic acid supplemented with a cold nucleotide standard used for UV-shadowing (10 mM GDP and 10 mM GTP) and spotted onto PEI-TLC plates (Macherey-Nagel). TLC was performed in 0.5 M KH_2_PO_4_ pH 3.5 buffer, the plates were dried, samples were cut into sections (guided by UV shadowing), and ^3^H radioactivity was quantified by scintillation counting in Optisafe-3 (Fisher) scintillation cocktail. Conversion of the substrate to product was quantified as described earlier (38).

### Enzymatic assays with E. coli RelA.

Biochemical assays utilized *in vitro* translation (92) and stringent response (38) systems from E. coli purified components. Experiments were performed in HEPES-Polymix buffer with either 5 mM Mg^2+^ (for enzymatically assembled initiation complexes) or 15 mM Mg^2+^ [for vacant 70S, as well as nonenzymatically assembled poly(U)-programmed ribosomes] (92, 93). A detailed description of the preparation of biochemical components can be found in the supplemental material.

Poly(U)-programmed system. For the poly(U)-programmed system, a mixture containing 0.5 μM 70S, 2 μM poly(U) (Sigma), 2 μM tRNA^Phe^ (ChemBlock), 0.1% (wt/vol) Pluronic F-127, and 100 μM ppGpp was preincubated for 2 min at 37°C, followed by the addition of 30 to 100 nM RelA and 300 μM [^3^H]GDP (Hartman or American Radiolabeled Chemicals), followed in turn by incubation for an additional 2 min at 37°C. After that the reaction was started by the addition of ATP to the final concentration of 1 mM, and time point samples (5 μl) were taken, quenched with formic acid, and analyzed by TLC.

Initiation complex system. For the initiation complex system, a mixture containing 0.1 μM RelA, 0.5 μM initiation complex, 2 μM tRNA^Phe^, 300 μM [^3^H]GDP, and 100 μM ppGpp was preincubated at 37°C for 2 min, and then the reaction was started by the addition of 1 mM ATP, and time point samples (5 μl) were taken, quenched with formic acid, and analyzed by TLC.

### GTPase assays with E. coli EF-G.

For the GTPase assays with E. coli EF-G, reaction mixtures containing 0.5 μM 70S, 0.1 μM EF-G, and 0.1% Pluronic F-127 (Sigma) in HEPES-Polymix (5 mM Mg^2+^) were preincubated for 2 min at 37°C prior to the addition of 300 μM [^3^H]GTP substrate (Hartman); time point samples (5 μl) were then taken, quenched with formic acid, and analyzed by TLC (see above).

### Growth assays in a 96-well plate format.

Defined MOPS medium (95) was supplemented with 0.4% (wt/vol) glucose as the carbon source and the full set of 20 amino acids. The amino acid set was added either at 25 μg/ml each (MOPS_aa25_) or serine was added at 400 μg/ml and the 19 other amino acids were added at 40 μg/ml (MOPS_aa40Ser400_). LB Lennox medium (Becton Dickinson) was prepared according to the manufacturer's instructions, but instead of autoclaving, the medium was filter sterilized using 0.2-μm-pore-size filters (96). In accordance with earlier recommendations for handling the ppGpp^0^ strain to avoid revertants (58), the starter cultures for all three strains (i.e., the wild-type, Δ*relA*, and ppGpp^0^ strains) were prepared as follows. A thick suspension was made in the medium of interest using several colonies from a fresh overnight LB agar plate, which was then diluted to a starting OD_600_ within 0.001 to 0.025. Growth (OD_600_) was monitored in flat-bottom, 96-well plates, with 100 μl of the resulting cell suspension per well. Uninoculated medium served as a negative control and a blank. The outer wells were not used and were filled with water to counteract evaporation. The plates were then covered with prewarmed (to avoid condensation) lids, followed by incubation at 37°C with shaking.

### Growth inhibition of E. coli by bacteriostatic antibiotics.

Overnight cultures were pregrown at 37°C with aeration (200 to 220 rpm) in MOPS medium supplemented with 0.4% glucose and amino acids (25 μg/ml), diluted 100-fold into 20 ml of fresh medium in 100-ml flasks, and grown until the samples reached an OD_600_ of 0.5. Antibiotics were then added at various concentrations, and the growth was monitored using OD_600_ as a readout. Growth inhibition was calculated as the increase in OD_600_ after 1 h of antibiotic treatment compared to the untreated control culture. Relative growth was calculated as follows: (OD_600_ treated – initial OD_600_)/(OD_600_ untreated – initial OD_600_).

### Killing of E. coli by bactericidal antibiotics.

Bactericidal antibiotic killings were performed as per Kaldalu et al. (96) using ampicillin at 200 μg/ml, imipenem at 4 μg/ml, and norfloxacin at 5 μg/ml. The general experimental setup is outlined in Fig. S1 in the supplemental material). Overnight cultures were grown at 37°C with aeration (200 to 220 rpm) either in MOPS medium with 0.4% glucose and amino acids at the indicated concentrations or in filtered LB medium, diluted 100-fold into the corresponding fresh medium, and grown until reaching an OD_600_ of 0.5. Pretreatment antibiotics were then added at the indicated concentrations, and the cells were incubated at 37°C with aeration for an additional 30 min. Next, the culture was challenged by the bactericidal antibiotics ampicillin (200 μg/ml), norfloxacin (5 μg/ml), or ampicillin and imipenem (200 and 4 μg/ml, respectively), and 10 μl aliquots were removed for colony counts. CFU were determined at time points by making five to six 10-fold dilutions in phosphate-buffered saline at room temperature on a sterile 96-well plate and dropping 5 μl from each dilution on an LB agar plate. Upon absorption of the spotted culture, the plates were incubated at 30°C or 37°C overnight, followed by colony counting using the most dilute droplet in which colonies were still separated well enough to enable reliable counting. We observed no differences in colony counts between overnight incubation at 30 or 37°C; however, the latter temperature was preferred since it resulted in smaller colonies and therefore yielded more precise colony counts. The surviving fraction was expressed in relation to the CFU count of the starter culture prior to the addition of bactericidal antibiotic. The data are presented as geometric means, and error bars indicate the standard errors of the mean.

### Nucleotide measurements.

B. subtilis BSB1 strain cells were grown in MOPS buffered medium optimized for B. subtilis according to the recommendations of Libor Krásný (unpublished data): 50 mM MOPS (pH 7.0), 1 mM (NH_4_)_2_SO_4_, 1 mM KH_2_PO_4_, 2 mM MgCl_2_, 2 mM CaCl_2_, 50 mM MnCl_2_, and 5 mM FeCl_3_, supplemented with 0.4% (wt/vol) glucose as a carbon source, as well as the full set of 20 amino acids, each at 25 μg/ml. The liquid culture was started from a fresh overnight LB agar plate using several colonies to make a thick suspension in 1 ml of MOPS medium. From that suspension, 190 ml of warm medium in a 1-liter flask was inoculated so that the final OD_600_ was 0.02. During OD_600_ measurement and inoculation, care was taken to use cells from the suspension, avoiding cell clumps. Cultures were then grown at 37°C with aeration (200 to 220 rpm) until the OD_600_ reached 0.5. Next, the culture was split into 45-ml portions (including untreated control culture), and treated cultures received antibiotics at the indicated concentrations. In experiments with thiostrepton, Pluronic F-127 was added at a 0.1% (wt/vol) final concentration (using 20% stock in DMSO), including untreated control culture. After 30 min at 37°C and 200 to 220 rpm, 45 ml was sampled for nucleotide determination by pouring on ice-cold formic acid at final 1 M, and the samples were snap-frozen in liquid nitrogen.

E. coli BW25113 strain cultures grown in MOPS buffered medium were prepared slightly differently than those for B. subtilis. Specifically, we followed the recipe of Neidhardt et al. (95); glucose was supplemented at 0.4% (wt/vol), and 20 amino acids were added at final concentrations of 25 μg/ml each. A 1 ml culture was started from a single colony from a freshly streaked LB agar plate. After overnight incubation at 37°C with aeration (200 to 220 rpm), the cells were 100-fold diluted into warm fresh medium and grown until reaching an OD_600_ of 0.5. Then, 10 ml of culture was removed for nucleotide determination by pipetting samples onto ice-cold formic acid and then quick-freezing them in liquid nitrogen.

In the case of E. coli, mupirocin was used at a concentration that reduces the growth rate by half (70 μM, 35 μg/ml) (see “Growth inhibition of E. coli by bacteriostatic antibiotics” for additional details). B. subtilis is dramatically more sensitive to mupirocin (see Fig. S2 in the supplemental material). The antibiotic does not slow B. subtilis growth in a concentration-dependent manner. Instead, at low mupirocin concentrations growth is transiently inhibited, resulting in a prominent lag phase. Therefore, for B. subtilis we used a concentration that inhibits growth for 1 h, which is potent enough to induce stringent response without a permanent inhibition of growth (70 nM, 0.035 μg/ml).

Nucleotide samples were prepared and analyzed essentially as described earlier for nucleotide measurements (97) using an Agilent 1100 system with Phenomenex SecurityGuard cartridges and a Phenomenex Sphereclone SAX column (5 ∝m, 4.6 by 150 mm). The identity of nucleotide peaks was confirmed by (i) spiking in nucleotide standards to experimental samples and (ii) comparing the spectral properties and retention times of peaks to those of nucleotide standards. The extraction efficiencies were calculated by using spiked-in standards.

## Supplementary Material

Supplemental material
