# Supplementary material for: Subinhibitory Concentrations of Bacteriostatic Antibiotics Induce relA-Dependent and relA-Independent Tolerance to β-Lactams
Source: Antimicrob Agents Chemother. 2017 Mar 24;61(4):e02173-16. doi: 10.1128/AAC.02173-16 (PMC5365698; doi:10.1128/AAC.02173-16)
Supplement: Supplemental material [file supp_61_4_e02173-16__index.html]

Supplemental material 

# Subinhibitory Concentrations of Bacteriostatic Antibiotics Induce *relA*-Dependent and *relA*-Independent Tolerance to β-Lactams

## Supplemental material

- Supplemental file 1 -

  Supplemental material

  PDF, 463K
