# Supplementary material for: Subinhibitory Concentrations of Bacteriostatic Antibiotics Induce relA-Dependent and relA-Independent Tolerance to β-Lactams
Source: Antimicrob Agents Chemother. 2017 Mar 24;61(4):e02173-16. doi: 10.1128/AAC.02173-16 (PMC5365698; doi:10.1128/AAC.02173-16)
Supplement: Supplemental material [file AAC.02173-16_zac004176020s1.pdf]

Supplemental Material

Sub-inhibitory concentrations of bacteriostatic antibiotics induce *relA*-dependent and *relA*-independent tolerance to β-lactams

Pavel Kudrin[1,⌘], Vallo Varik[1,2,3⌘], Sofia Raquel Alves Oliveira[1], Jelena Beljantseva[1], Teresa Del Peso Santos[2,3], Ievgen Dzhygyr[2,3], Dominik Rejman[4], Felipe Cava[2,3], Tanel Tenson[1], Vasili Hauryliuk[1,2,3,*]

University of Tartu, Institute of Technology, Tartu, Estonia[1]; Department of Molecular Biology, Umeå University, Umeå, Sweden[2]; Laboratory for Molecular Infection Medicine Sweden (MIMS), Umeå University, Umeå, Sweden[3]; Institute of Organic Chemistry and Biochemistry, Czech Academy of Sciences v.v.i., Flemingovo nám. 2, 166 10 Prague 6, Czech Republic[4]

Running head: *relA*-dependent and *relA*-independent β-lactam tolerance

[*] address correspondence to Vasili Hauryliuk, vasili.hauryliuk@umu.se

[⌘] P.K. and V.V. contributed equally to this work

Supplementary Methods

Preparation of thiostrepton stock solutions

Several crystals of thiostrepton (Tocris Bioscience, validated by mass spectrometry) dissolved in ≈300 μl of 100% DMSO (Sigma Aldrich) and the concentration (≈600 mM) is measured by absorbance at 280 nm with ε = 0.027 $\mu M^{-1}cm^{-1}$. DMSO stock is then used to prepare the working stock in 1x Hepes:polymix buffer (25 mM Hepes-KOH pH 7.5, 15 mM $MgCl_2$, 0.5 mM CaCl, 95 mM KCl, 5 mM NH_4Cl, 8 mM putrescine, 1 mM spermidine, 5 mM K_3PO_4 pH 7.3 and 1 mM DTT (1)) supplemented 0.1% Pluronic F-127 (Sigma Aldrich) as follows. Thiostrepton stock in DMSO was mixed with 20% (v/w) Pluronic F-127 in DMSO and 1x Hepes:polymix buffer was gradually added to final volume to achieve the final concentration of 12.5 μM. The final DMSO concentration should be kept below 3% and all the preparations should be done at room temperature to avoid precipitation. Working stock was briefly centrifuged (21,000 rcf, 5 min) at room temperature in order to remove any traces of precipitated thiostrepton, supernatant transferred into a new tube and the final concentration of thiostrepton re-measured using absorbance at 280 nm using 1x Hepes:polymix supplemented with 3% DMSO and 0.1% Pluronic F-127 as a blank reference.

Preparation of ppGpp

ppGpp was produced enzymatically using either Rel_{Seq} enzyme from *Streptococcus equisimilis* (2) or RelQ enzyme from *Enterococcus faecalis* (3) essentially as described in Mechold et al. (4). Reaction mixture containing 5 mM GDP, 10 mM ATP, 15 mM $MgCl_2$, 30 mM Tris-HCl (pH 8.0), 100 mM NaCl in MilliQ H_2O was preincubated for 5 min at 37 °C followed by the addition of 50 μM Rel_{Seq} or 5 μM RelQ. Since Rel_{Seq} is strongly inhibited by free Mg^{2+} ions, therefore the total nucleotide concentration (GDP + ATP) should be equal to that of $MgCl_2$; in the case of using RelQ free Mg^{2+} is not a concern since due to RelQ's insensitivity. After 2 hours of incubation at 37 °C with constant shaking, nucleotides were extracted by the addition of acidic phenol (pH 4.5) (Amresco); directly after phenol addition to the reaction (1:1), the mixture was subjected to vigorous vortexing followed by centrifugation (16k rcf, 4 °C, 30 min) in order to separate the phases. To completely remove the traces of phenol this step was repeated. Upper phase was collected and applied onto MonoQ™ 5/50 GL (GE Healthcare) column on equilibrated in buffer **A** (2.5 mM Tris-HCl (pH 8.0), 0.5 mM EDTA and 0.5 mM LiCl in MilliQ H_2O). Nucleotides were separated by gradient elution increasing concentration of LiCl up to 2 M. We have collected the ppGpp peak that eluts at ≈250 mM LiCl, and the nucelotide was precipitated by the addition of 1 M LiCl (f.c.) and 3 volumes of cold 96% EtOH followed incubation either at -20 °C (overnight) or at -80 °C (2 hours). The precipitate was vortexed, transferred to centrifugation tubes and pelleted at 16k rcf, 4 °C for 30 min. Supernatant was discarded, the pellet washed with -20 °C 70% EtOH in order to remove traces of LiCl and the pellet (ppGpp) dissolved in MilliQ H_2O. The concentration of ppGpp was determined with extinction coefficient E_a = 13,600 $\mu M^{-1}cm^{-1}$ at 252 nm.

Preparation of RelA and EF-G

C-terminally 6His-tagged RelA (5) was overexpressed in *E. coli* BL21(DE3) from pET28a plasmid; a colony from overnight grown on LB/Kanamycin agar plate was inoculated into 3 ml of LB media and grown shaking overday at 37 °C in the presence of 25 µg/ml Kn to OD_{600}=0.5. After that the culture was transferred into 200 ml of fresh pre-warmed 2x YT media (with 25 µg/ml Kn) and grown shaking at 37 °C to OD_{600}=0.5 followed by the induction of RelA overexpression by adding 1 mM IPTG (Sigma). Straightly after induction the temperature was decreased to 30 °C and bacteria was left to grow for 1.5 hours. Afterwards the bacterial cell mass was collected by centrifugation. For the lysis step bacteria were resuspended in a buffer, consisting of 25 mM Tris (pH 7.5), 2 mM $MgCl_2$ and 1 mM 2-mercaptoethanol (βME) with the addition of 100 µM PMSF and 1 U/ml DNase I (Thermo Scientific). Cells were lysed with Stansted SPCH-10 homogenizer and the lysate centrifuged with following application for Ni-NTA affinity chromatography in GE Healthcare HisTrap HP column on ÄktaPrimePlus (GE Healthcare). The running buffer consisted of 25 mM Tris (pH 7.5-8), 1 M KCl, 350 mM NaCl, 2 mM $MgCl_2$, 5 mM imidazole, 1 mM βME while in the elution buffer the concentration of imidazole was increased to 300 mM. RelA-correspondent peak was collected and the protein concentrated using Amicon Ultra-15 (Millipore) centrifugal filter devices with a 50 kDa cut-off. The final preparations were aliquoted by 15 µl, shock-frozen with liquid nitrogen and stored at –80 °C in buffer containing 25 mM HEPES-KOH, pH 7.5, 0.7 M KCl, 2 mM DTT, 15 mM $MgCl_2$ and 10% glycerol.

N-terminally 6His-tagged EF-G protein was overexpressed in *E. coli* BL21(DE3) from pQE30 plasmid provided by J. Remme (6). The overnight-grown culture was transferred into 200 ml of fresh 2x YT media (with 100 µg/ml Amp) and grown shaking at 37 °C to OD_{600}=0.5 followed by the induction of EF-G overexpression by adding 1 mM IPTG (Sigma). After the induction the bacteria were left to grow shaking for 2 more hours at 37 °C with the cell mass being collected by centrifugation in the end. Cells were lysed with Stansted SPCH-10 homogenizer in buffer **A** (50 mM Tris-HCl pH 7.5, 10 mM $MgCl_2$, 200 mM PMSF and DNase I) and clarified by centrifugation at 40k rpm in Ti 50 rotor for 40 min (Beckman & Coulter). Clarified lysate was loaded on HisTrap HP Ni-NTA affinity chromatography column (GE Healthcare) and the protein was eluted by a gradient of buffer **B** (**A** supplemented with 300 mM imidazole). EF-G was concentrated and stored –80 °C in buffer containing 25 mM HEPES-KOH, pH 7.5, 0.7 M KCl, 2 mM DTT, 15 mM $MgCl_2$ and 10% glycerol.

fMet-tRNA^{fMet} purification on BD sepharose ion-exchange column

Preparation was done essentially as described in (7). The starting material, deacylated *E. coli* $tRNA_i^{fMet}$ was purchased from Chemblock. The aminoacylation and formylation reaction mixture containing 100 µM deacylated $tRNA_i^{fMet}$, 300 µM ^{3}H-methionine (Perkin Elmer), 1 µM *E. coli* MetRS, 1 µM *E. coli* formylase (FMT), 1 mM THF, 2 mM ATP, 3 mM PEP, 0.05 g/l phosphokinase, 0.01 g/l yeast pyrophosphatase (100 U/mg, Sigma-Aldrich), 0.002 g/l myokinase, 3 mM β-mercaptoethanol, 50 mM HEPES, 50 mM KCl and 10 mM $MgCl_2$ was incubated at

37 ˚C for 30 min and the reaction was stopped by adding 10 mL of cold buffer **A** (20 mM NaOAc pH 5.1, 1 mM EDTA, 5 mM BME). 100 ml BD Sepharose column was equilibrated with 5 CV of buffer **A** at room temperature. The reaction mixture diluted in 10 mL of buffer **A** was loaded at speed of 1 ml/min, equilibrated with buffer **A** until the baseline, and ^{3}H-fMet-tRNA$_i^{fMet}$ was eluted with a two-step gradient of the buffer **B** (**B** = **A** + 1M NaCl): 1.5 CV 0-50% (**B**) followed by 2.5 CV 50-100% (B). The fractions containing fMet-tRNA$_i^{fMet}$ peak (≈95% **B**) (identified by scintillation counting) was collected and precipitated with 3 volumes of EtOH (95%) and 300 mM KOAc at -20°C overnight. The precipitate was centrifuged at 20000 rpm in Ti45 rotor (Backman & Coulter) for 40 min, the pellet dissolved in 4 ml in KOAc 5 mM (pH 5.1) and re-precipitated again with KOAc and EtOH as stated above for 30 min at -80°C. Precipitate was centrifuged at 21,000 rcf for 15 min in the bench-top centrifuge. The pellet was washed with 500 µl EtOH 70%, dried at room tepmeratrue and dissolved in 5 mM KOAc (pH 5.1) to yield a 100 µM stock solution. Concentration and charging levels of fMet-tRNA$_i^{fMet}$ were determined spectrophotometrically (1 AU_{260} = 40 ng/µl RNA, Mw tRNA = 25 kDa) and by scintillation counting using ^{3}H-methionine used for charging reaction as a standard.

Preparation of ribosomal initiation complexes

Wild type MRE600 *E. coli* and A1067U (provided by J. Remme) 70S ribosomes were prepared as per (7). Model mRNA(MF) 5'-GGCAAGGAGGUAAAAAUGUUCAAA-3' was purchased from Sigma-Aldrich. To form the initiation complexes, 8 µM 70S ribosomes, 3.1 µM IF1, 4.4 µM IF2, 3.1 µM IF3, 12 µM mRNA, 12 µM [^{3}H]fMet-tRNA^{fMet}, 1 mM GTP were mixed in Hepes:polymix buffer (final reaction volume 500 µl; 2 mM DTT and 5 mM Mg^{2+}). The mixture was incubated at 37°C for 30 minutes, chilled on ice for 5 minutes, Mg^{2+} concentration adjusted to 15 mM and the reaction mixture was loaded on 400 µl sucrose cushion (1.1 M sucrose, 1x Hepes:polymix, 15 mM Mg^{2+}) and centrifuged at 45,000 rpm in TLS-55 rotor (Beckman & Coulter) for 2 hours, supernatant discarded and the pellet dissolved in 200 µl of polymix buffer (5 mM Mg^{2+}), aliquoted, snap-frozen with liquid nitrogen and stored at -80 ˚C. Ribosomal concentration was determined by diluting 2 µl of IC 2000 times in water and measuring the absorbance at 260 nm: 1 AU_{260} = 23 nM 70S. [^{3}H]fMet-tRNA^{fMet} concentration was determined by scintillation counting. IC occupancy was calculated as ratio between fMet-tRNA$_i^{fMet}$ and 70S concentrations.

Isolation of peptidoglycan and UPLC analysis

Peptidoglycan of *E. coli* was isolated as described previously (8), with minor modifications. *E. coli* strains were grown in MOPS media (9), supplemented with 0.4% glucose and 25 µg/ml of 20 amino acids. 600 ml of media was inoculated from 1 ml of overnight culture and grown at 37 °C with vigorous aeration until OD_{600} = 0.5, then the corresponding antibiotics were added for further incubation. Cells were harvested by 10 min at 5000 g at room temperature, washed with 10 ml PBS, harvested again and snap frozen in liquid nitrogen. Pellets were re-suspended in 3 ml of PBS, added to an equal volume of 10% SDS in a boiling water bath and vigorously stirred for 3 h, then stirred overnight at 37

°C. The insoluble fraction (peptidoglycan) was pelleted at 60000 rpm, 15 min, 20 °C using a TL100 Beckman rotor in an Optima MAX-TL ultracentrifuge (Beckman Coulter), and then washed 3 times with MQ-water. Samples were digested for 1 h at 60 °C with pronase E (100 µg/ml) in 10 mM Tris-HCl pH 7.5 and NaCl 0.06% (w/v), to remove Braun's lipoprotein. The reaction was heat-inactivated by adding SDS to a final concentration of 1% (w/v) and boiling for 10 min in a water bath. SDS was then removed by washing 3 times with MQ-water. Purified peptidoglycan was re-suspended in 100 µl of 50 mM $NaPO_4$ buffer, pH 4.9 and digested overnight at 37 °C with 100 µg/ml muramidase. The digestion was stopped by 15 min incubation in a boiling water bath, and coagulated proteins were removed by 10 min centrifugation at 14,000 rpm. The supernatants, containing the digested muropeptides, were reduced by adding 15 µl 0.5 M sodium borate pH 9 and 5 µl 2M sodium borohydride and incubating at room temperature for 30 min. Finally, samples were adjusted to pH 3.5 with phosphoric acid.

Muropeptides were separated in a linear gradient of 50 mM $NaPO_4$, pH 4.35 to 50 mM $NaPO_4$, pH 4.95, 15% methanol (v/v) in 20 min using a 0.25 ml/min flow on C18 column (150x21, 100Å pore size) column using Acquity UPLC (Waters) (**Supplementary Figure 9A**). Peptides were detected at Abs 204 nm. The relative abundance of the individual muropeptides was calculated as the % of the total area. The values are the means of two or three independent experiments. The analysis resolves monomeric (M), dimeric (D) and trimeric (T) muropeptides, referred to as per Glauner and colleagues (1988) (10): M3: disaccharide tripeptide (GlcNAc-MurNAc-L-Ala-D-Glu-g-*meso*DAP); M4: disaccharide tetrapeptide (GlcNAc-MurNAc-L-Ala-D-Glu-g-*meso*DAP-D-Ala); D33: LD-crosslinked (DAP-DAP) dimer tetrasaccharide hexapeptide; D34: LD-crosslinked (DAP-DAP) dimer tetrasaccharide heptapeptide; D43: DD-crosslinked (D-Ala-DAP) dimer tetrasaccharide heptapeptide; T444: DD-crosslinked trimer hexasaccharide dodecapeptide. Braun's lipoprotein bound monomer (M3-Lpp): GlcNAc-MurNAcL-Ala-D-Glu-g-*meso*DAP-e-L-Lys-L-Arg; and dimer (D33L): GlcNAc-MurNAcL-Ala-D-Glu-g-*meso*DAP-(e-L-Lys-L-Arg)-g-D-Glu-L-Ala-MurNAc-GlcNAc. The Thr-Arg dipeptide corresponds to the C-terminal dipeptide of *E. coli* Braun's lipoprotein. Identities of *E. coli* LC muropeptides on UPLC profile were identified as per (10) and confirmed genetically (11, 12).

Supplementary Results

Ampicillin's molecular target is penicillin-binding proteins, PBPs (13) – DD-transpeptidases generating crosslinks between D amino acids in peptidoglycan sacculus (14). While the DD crosslinks form the majority of the crosslinks, another type – LD, i.e. between L and D stereocenters of diaminopimelic acid (DAP) – are formed by ampicillin-insensitive LD-transpeptidases, Ldts (14).

HPLC analysis of peptidoglycan composition revealed that LD crosslinks between DAP moieties in the third position are significantly enriched in both wild type and relaxed strains upon antibiotic pre-treatment, especially in the case of mupirocin and trimethoprim combination (**Supplementary Figure 9AB**). Remodeling the cell wall depends on both *de novo* synthesis of peptidoglycan and remodeling of the existing one. Therefore we performed a kinetic analysis of DAP-DAP accumulation in relaxed strain exposed to either to mupirocin alone or mupirocin together with trimethoprim. In the latter case the fraction of DAP-DAP crosslink increases more than six times from 1.8 % prior to antibiotic challenge to 11% after five and a half hours (**Supplementary Figure 9C**).

However, a significant increase in the DAP-DAP crosslink (two and a half times) is observed upon mupirocin challenge of relaxed strain without the concomitant increase in ampicillin tolerance (**Supplementary Figure 9D**), indicating that the relationship between peptidoglycan remodeling and ampicillin tolerance is unlikely to be direct. To directly disprove the causal connection between accumulation of LD crosslinks and antibiotic-induced ampicillin tolerance we took advantage of bactericidal β-lactam antibiotic imipenem which is a potent Ldt inhibitor (15): if accumulation of the LD crosslinks is, indeed, essential for *E. coli* survival upon PBP inhibition by ampicillin, then a combination of imipenem and ampicillin should result in bacterial lysis. However, trimethoprim and mupirocin showed the same *relA*-independent protective effect in killing assays employing simultaneous challenge by imipenem and ampicillin (**Supplementary Figure 9E**). This suggests that, first, the pretreatment leads to tolerance to β-lactams in general rather than specifically to ampicillin, and, second, that accumulation of the LD crosslinks is not the cause of the effect. Finally, we used an *E. coli* strain lacking functional Ldt genes *ycbB* (*ldtD*) and *ynhG* (*ldtE*) and therefore unable to form DAP-DAP crosslinks (16). Pre-treatment with trimethoprim combined with either chloramphenicol or tetracycline protects this strain from ampicillin, directly confirming that LD crosslinks are not necessary for the effect (**Supplementary Figure 9E**).

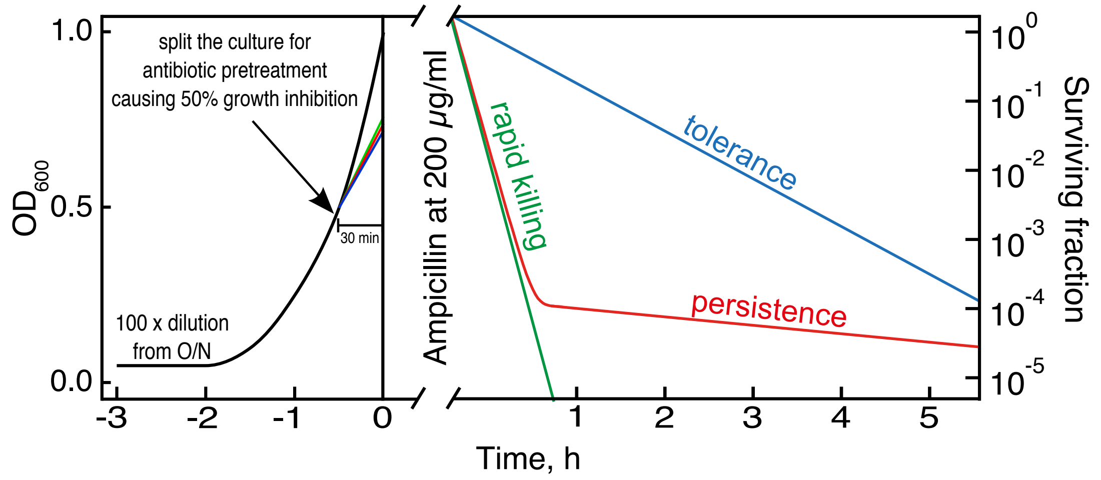

Supplementary Figure 1 | Experimental strategy for bactericidal antibiotic killings.

At OD_{600} of 0.5 bacteriostatic antibiotics were added at concentrations that reduce the growth rate two times and incubated for additional 30 minutes. After that bactericidal antibiotic ampicillin was added to final concentration of 200 μg/ml and the time course of antibiotic killing was followed by determining the surviving fraction using LB plating and colony count. The slower killing phase or plateau is defined as persistence, and slower killing of the bacterial population as a whole is defined as antibiotic tolerance. If not stated otherwise, the experiments were performed at 37°C in MOPS media supplemented with 0.4% glucose and 25 μg/ml amino acids using BW25113 *E. coli* strains. The antibiotics were used at concentrations causing growth rate reduction of two times.

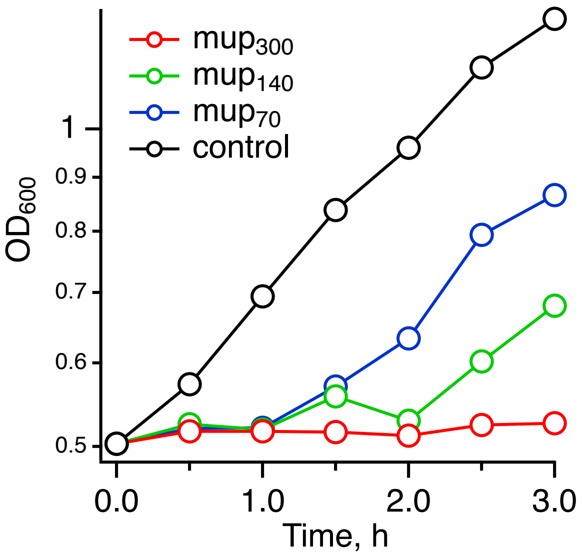

Supplementary Figure 2 | Instead of gradual inhibition of growth, mupirocin induces a pronounced lag phase in culture of *B. subtilis*.

The growth experiments were performed at 37°C in MOPS medium supplemented with 0.4% glucose and 25 µg/ml of each amino acid using BSB1 *B. subtilis* wild type strain. Cells were pre-grown to OD_{600} of 0.5 prior to addition of mupirocin and the consequent growth was followed. Antibiotic concentrations in nM are indicated on the figures, e.g. mup_{70} corresponds to 70 nM mupirocin.

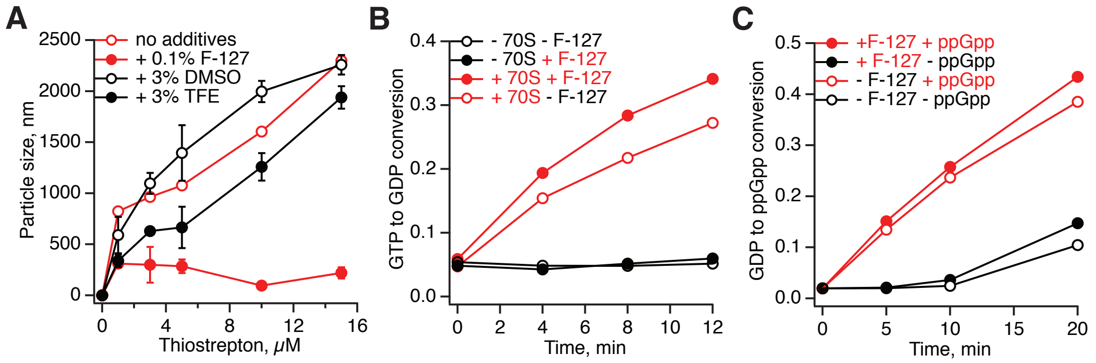

Supplementary Figure 3 | Pluronic F-127 solubilizes thiostrepton and does not interfere with EF-G and RelA activity.

(**A**) Effect TFE (filled black circles), DMSO (empty black circles), F-127 (filled red circles) on thiostrepton solubility as judged by the particle size measured with DLS. Analysis has been prepared in triplicates and the error bars indicate standard deviation. (**B**) EF-G GTP hydrolysis activity is insensitive to the addition of 0.1 % of Pluronic F-127, either in the presence (red circles) or absence (black circles) of vacant wild type 70S ribosomes. (**C**) RelA ppGpp synthesis activity is insensitive to the addition of 0.1 % of Pluronic F-127, either in the presence (red circles) or absence (black circles) of ppGpp.

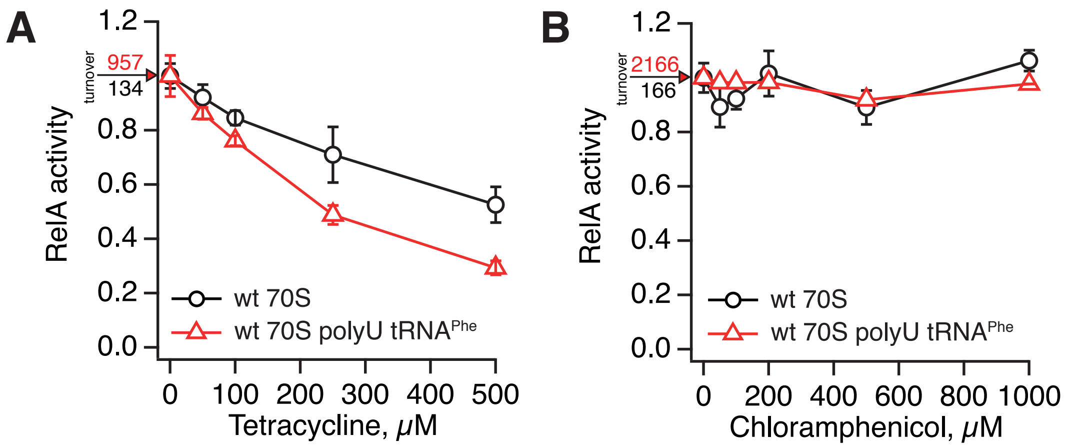

Supplementary Figure 4 | Tetracycline and chloramphenicol are poor and A-site tRNA insensitive inhibitors of RelA's enzymatic activity in the test tube.

Enzymatic activity of 30 nM RelA was assayed in the presence of 0.5 μM 70S ribosomes, either vacant (empty circles) or programmed with 2 μM poly(U) mRNA and 2 μM deacylated $tRNA^{Phe}$ (filled circles). All experiments were performed in Hepes:polymix buffer (15 mM Mg^{2+} and 0.1% Pluronic F-127), 0.3 mM ^{3}H-GDP, 1 mM ATP and 100 μM ppGpp. (**A**) tetracycline and (**B**) chloramphenicol. Enzymatic activities (turnovers, ppGpp per RelA per minute) were normalized to that of the corresponding system in the absence of antibiotics. Error bars represent standard deviations of the turnover estimates by linear regression, each experiment was performed at least three times.

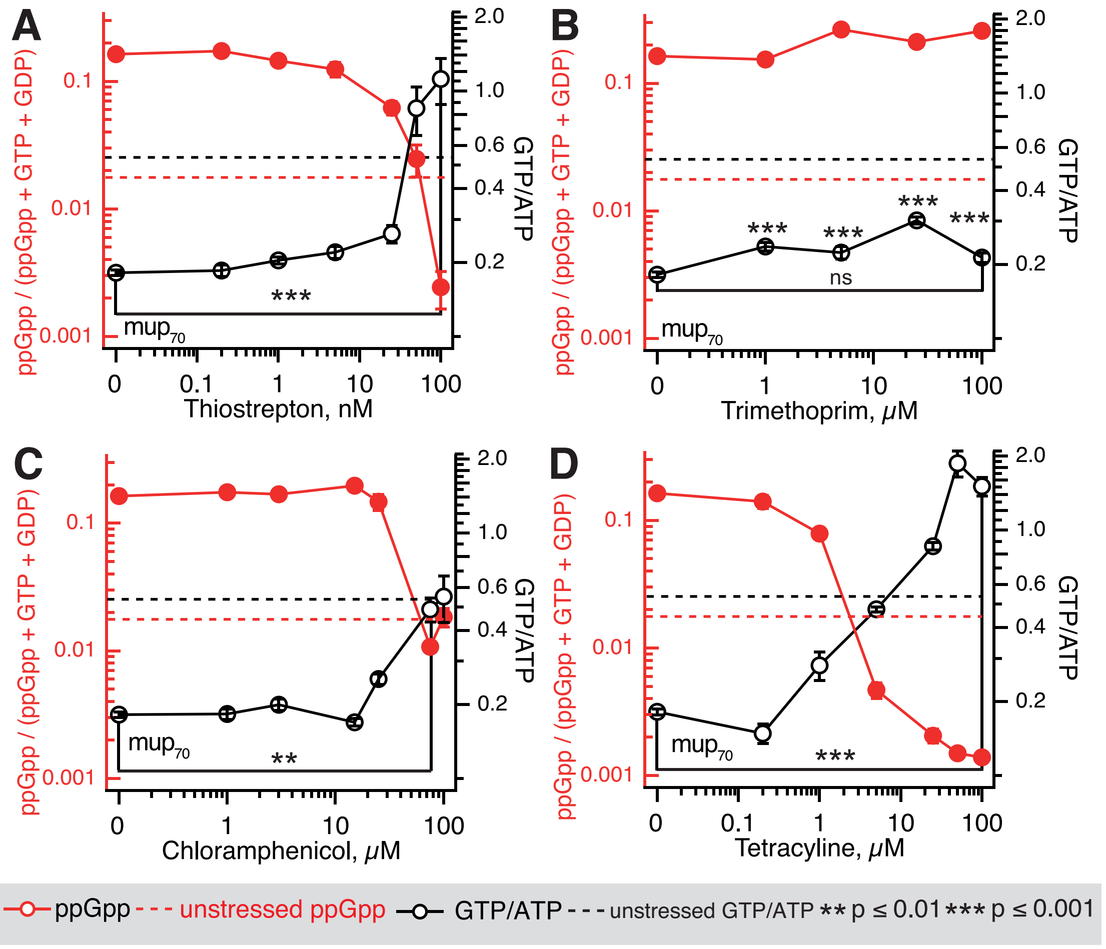

Supplementary Figure 5 | Antibiotics targeting translation cause concurrent inhibition of ppGpp production and increase in the GTP/ATP ratio in *B. subtilis* artificially starved for isoleucine by mupirocin.

Bacterial cultures were treated by addition of 70 nM mupirocin (designated by mup_{70}) combined with increasing concentrations of thiostrepton (**A**), chloramphenicol (**B**), tetracycline (**C**) or trimethoprim (**D**). 30 minutes after addition of antibiotics samples were collected and nucleotide levels determined by HPLC. Experiments were performed with BSB1 *B. subtilis* wild type strain grown at 37 °C in MOPS medium supplemented with 0.4% glucose and a full set of 20 amino acids at 25 μg/ml. ppGpp levels are calculated as a ppGpp fraction of combined GTP, GTP and ppGpp nucleotide pool, dashed red trace indicates the ppGpp levels in unstressed cells. Dashed black trace indicates the GTP/ATP ratio in unstressed cells. Error bars indicate standard error of the mean (3-5 biological replicates). As indicated by brackets, the P-values were calculated using the two-tailed Welch's t-test either in relationship to the untreated culture or within the titration series.

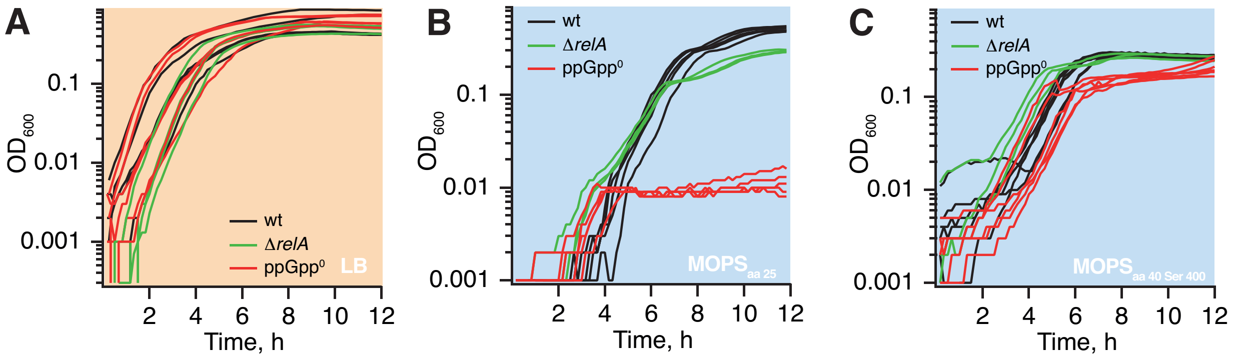

Supplementary Figure 6 | Growth wild type, Δ*relA* and ppGpp⁰ *E. coli* in LB and MOPS media.

E. coli wild type BW25113 as well as isogenic Δ*relA* and ppGpp⁰ (Δ*relA*Δ*spoT*) strains were grown on 96-well plate format (100 µl per well with fast shaking at 37°C) in filtered LB (**A**), and MOPS medium supplemented with 0.4% glucose and amino acids at either 25 µg/ml (**B**) or Serine at 400 and the rest at of the amino acids at 40 µg/ml (**C**).

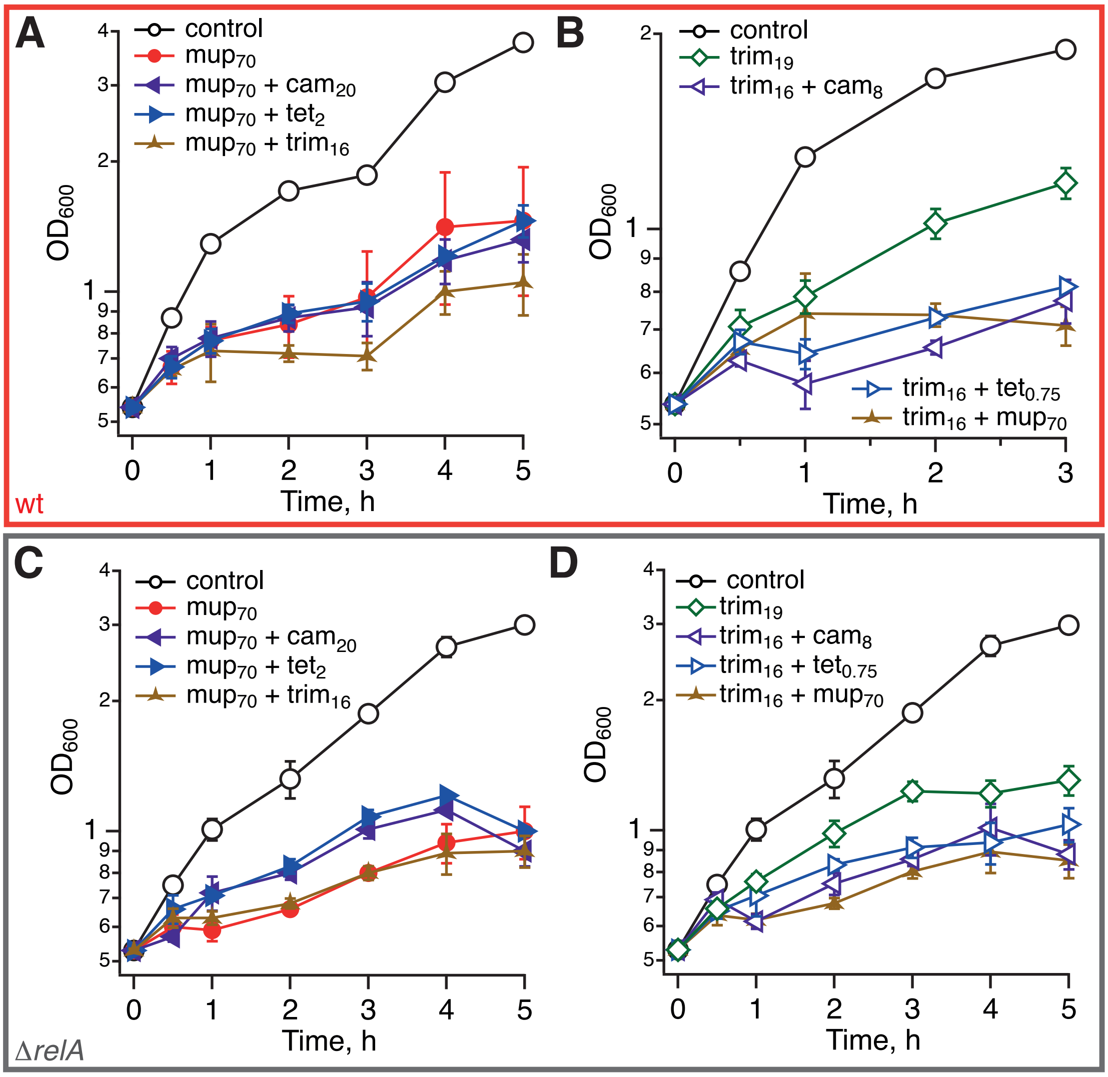

Supplementary Figure 7 | Effects of antibiotic combinations on BW25113 *E. coli* growth.

The growth experiments were performed at 37°C in MOPS medium supplemented with 0.4% glucose and 25 µg/ml of each amino acid using BW25113 *E. coli* wild-type strain (**A** and **B**) and an isogenic *relA* knock-out (Δ*relA*, **C** and **D**). Cells were pre-grown to OD_{600}=0.5, antibiotics (mup = mupirocin, cam = chloramphenicol, tet = tetracycline, trim = trimethoprim, + designates antibiotic combinations) were added, and the consequent growth was followed. Antibiotic concentrations in µM are indicated on the figures, e.g. mup_{70} + tet_2 designates treatment with 70 µM mupirocin and 2 µM tetracycline. Experiments were performed at least three times and error bars indicate standard error of the mean.

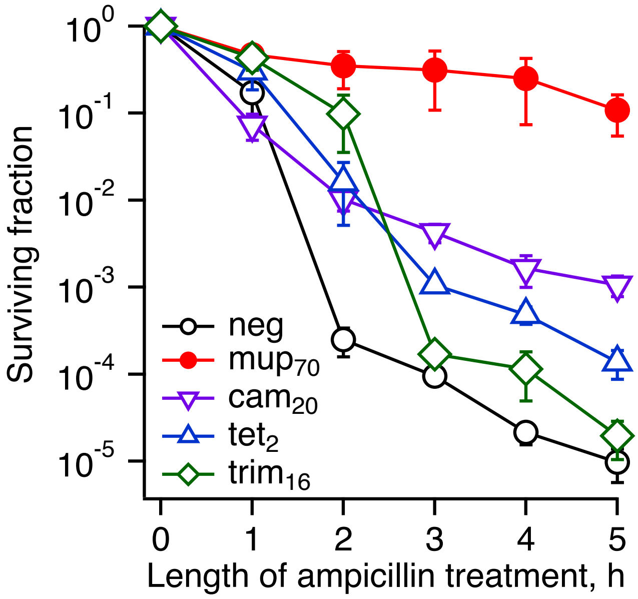

Supplementary Figure 8 | Effects of pre-treatment with individual antibiotics at growth suppressing concentrations on ampicillin tolerance of wild type BW25113 *E. coli*.

The antibiotics were used at concentrations causing complete inhibition of growth and maximum reduction in the ppGpp levels (20 µM chloramphenicol, 2 µM tetracycline and 16 µM trimethoprim), except for mupirocin (70 µM), which was used at a concentration causing a two-fold reduction of the growth rate and serves as a comparison for the results on **Figures 6A** and **6B**. Antibiotic concentrations in µM are indicated on the figures, e.g. mup_{70} designates pre-treatment with 70 µM mupirocin. The antibiotic pre-treatment was performed for 30 minutes at 37°C in MOPS medium supplemented with 0.4% glucose and 25 µg/ml of each amino acid, followed by addition of ampicillin to final concentration of 200 µg/ ml. The surviving fraction was determined by LB plating and colony count. Experiments were performed at least three times and error bars indicate standard error of the mean.

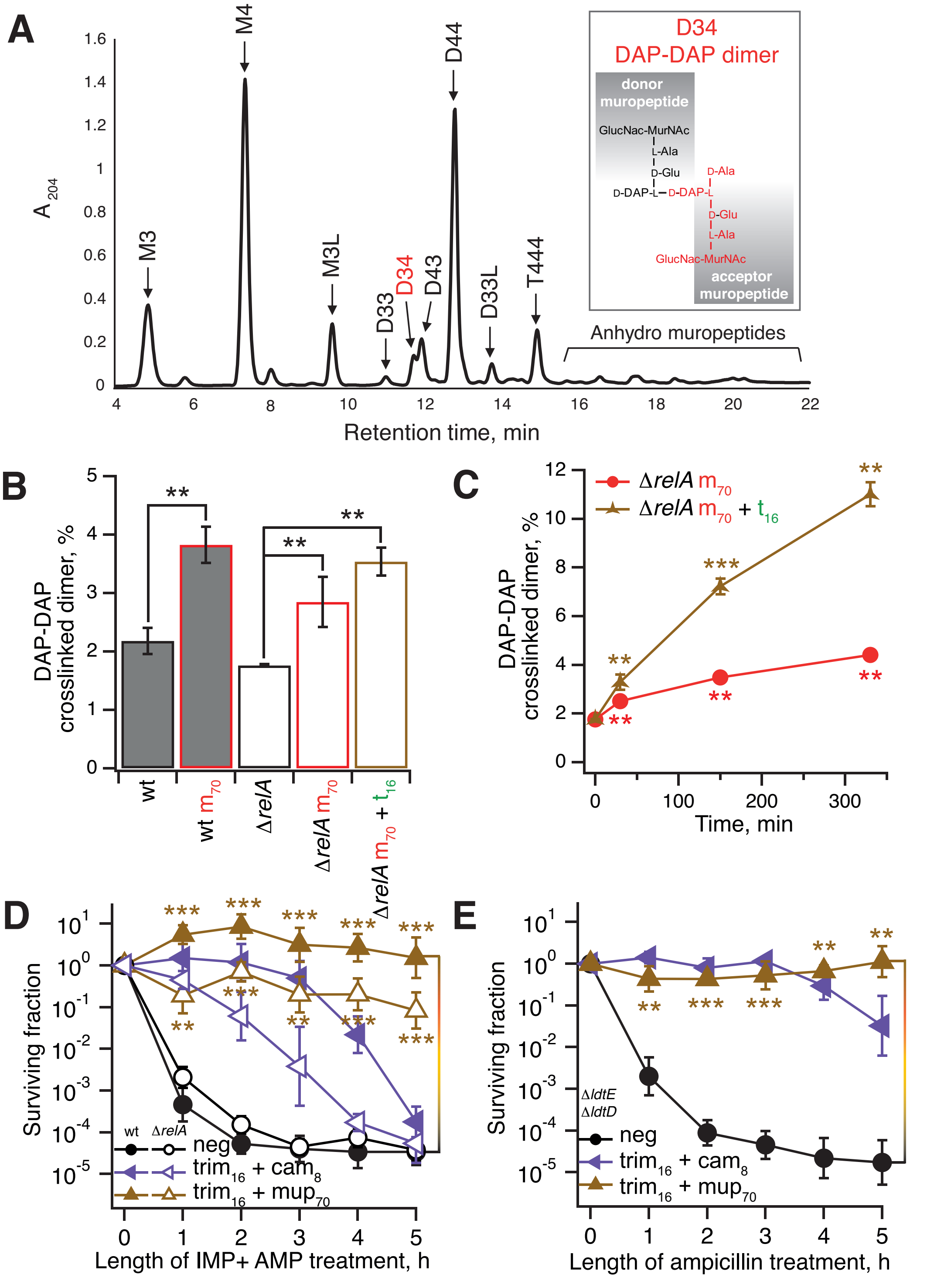

** $p \leq 0.01$ *** $p \leq 0.001$

Figure 9 | Accumulation of DAP-DAP crosslinks upon mupirocin and trimethoprim challenge is not responsible for β-lactam tolerance. (A) HPLC analysis of *E. coli* peptidoglycan composition was used to resolve monomeric (M), dimeric (D) and trimeric (T) muropeptides. DD-transpeptidases (PBPs) catalyze crosslinking between α-carboxyl of D-Ala in fourth position of the peptide moiety of the donor monomer and the ε-amine of mesoDAP of the acceptor monomer (D43, D44 and T444). Ampicillin-insensitive LD-

transpeptidases (Ldts) catalyze DAP-DAP between the L- and D-stereogenic centers of two mesoDAP (D33 and D34, see insert). (**B**) A 30 minute-long pre-treatment with mupirocin (m) alone and in combination with trimethoprim (t) resulted in accumulation of DAP-DAP crosslinks. (**C**) Longer exposure to antibiotics resulted in DAP-DAP crosslinked dipeptide becoming one of the dominant species. To probe the role of DAP-DAP crosslinked dipeptide in ampicillin resistance induced by mupirocin combined with trimethoprim, ampicillin (AMP, 200 μg/ml) killing was followed in conditions when DAP-DAP formation was abrogated either chemically by addition of saturating concentrations of bactericidal antibiotic Ldt inhibitor imipenem (IMP, 4 μg/ml) (**D**) or genetically by using Δ*ldtE* Δ*ldtD E. coli* strain (**E**). Antibiotics concentrations in μM are indicated on the figures, e.g. mup_{70} designates pre-treatment with 70 μM mupirocin. Error bars indicate standard deviation of three independent experiments. As indicated by brackets, the P-values were calculated using two-tailed Welch's t-test in relationship to killing time courses of untreated culture.

Supplementary references:

1. **Jelenc PC, Kurland CG.** 1979. Nucleoside triphosphate regeneration decreases the frequency of translation errors. Proc Natl Acad Sci U S A **76:**3174-3178.
2. **Hogg T, Mechold U, Malke H, Cashel M, Hilgenfeld R.** 2004. Conformational antagonism between opposing active sites in a bifunctional RelA/SpoT homolog modulates (p)ppGpp metabolism during the stringent response [corrected]. Cell **117:**57-68.
3. **Gaca AO, Kudrin P, Colomer-Winter C, Beljantseva J, Liu K, Anderson B, Wang JD, Rejman D, Potrykus K, Cashel M, Hauryliuk V, Lemos JA.** 2015. From (p)ppGpp to (pp)pGpp: Characterization of Regulatory Effects of pGpp Synthesized by the Small Alarmone Synthetase of Enterococcus faecalis. J Bacteriol doi:10.1128/JB.00324-15.
4. **Mechold U, Murphy H, Brown L, Cashel M.** 2002. Intramolecular regulation of the opposing (p)ppGpp catalytic activities of Rel(Seq), the Rel/Spo enzyme from Streptococcus equisimilis. J Bacteriol **184:**2878-2888.
5. **Knutsson Jenvert RM, Holmberg Schiavone L.** 2005. Characterization of the tRNA and ribosome-dependent pppGpp-synthesis by recombinant stringent factor from Escherichia coli. FEBS J **272:**685-695.
6. **Pulk A, Maivali U, Remme J.** 2006. Identification of nucleotides in E. coli 16S rRNA essential for ribosome subunit association. RNA **12:**790-796.
7. **Antoun A, Pavlov MY, Tenson T, Ehrenberg MM.** 2004. Ribosome formation from subunits studied by stopped-flow and Rayleigh light scattering. Biol Proced Online **6:**35-54.
8. **Cava F, de Pedro MA, Lam H, Davis BM, Waldor MK.** 2011. Distinct pathways for modification of the bacterial cell wall by non-canonical D-amino acids. EMBO J **30:**3442-3453.
9. **Neidhardt FC, Bloch PL, Smith DF.** 1974. Culture medium for enterobacteria. J Bacteriol **119:**736-747.
10. **Glauner B, Holtje JV, Schwarz U.** 1988. The composition of the murein of Escherichia coli. J Biol Chem **263:**10088-10095.
11. **Magnet S, Bellais S, Dubost L, Fourgeaud M, Mainardi JL, Petit-Frere S, Marie A, Mengin-Lecreulx D, Arthur M, Gutmann L.** 2007. Identification of the L,D-transpeptidases responsible for attachment of the Braun lipoprotein to Escherichia coli peptidoglycan. J Bacteriol **189:**3927-3931.
12. **Magnet S, Dubost L, Marie A, Arthur M, Gutmann L.** 2008. Identification of the L,D-transpeptidases for peptidoglycan cross-linking in Escherichia coli. J Bacteriol **190:**4782-4785.
13. **Zapun A, Contreras-Martel C, Vernet T.** 2008. Penicillin-binding proteins and beta-lactam resistance. FEMS Microbiol Rev **32:**361-385.
14. **Typas A, Banzhaf M, Gross CA, Vollmer W.** 2012. From the regulation of peptidoglycan synthesis to bacterial growth and morphology. Nat Rev Microbiol **10:**123-136.
15. **Mainardi JL, Hugonnet JE, Rusconi F, Fourgeaud M, Dubost L, Moumi AN, Delfosse V, Mayer C, Gutmann L, Rice LB, Arthur M.** 2007.

Unexpected inhibition of peptidoglycan LD-transpeptidase from Enterococcus faecium by the beta-lactam imipenem. J Biol Chem **282:**30414-30422.
16. **Sanders AN, Pavelka MS.** 2013. Phenotypic analysis of Eschericia coli mutants lacking L,D-transpeptidases. Microbiology **159:**1842-1852.
